# Sensor-Based Movement Quality Assessment and Biofeedback for Rehabilitation Exercise: A Scoping Review with Implications for Home-Based and Remote Rehabilitation

**DOI:** 10.3390/healthcare14162450

**Published:** 2026-08-07

**Authors:** Tao Mei, Yulong Wang, Wenze Xu, Xueke Liu, Liang Li

**Affiliations:** 1 China Institute of Sport and Health Science, Beijing Sport University, Beijing 100084, China; meitao@bsu.edu.cn (T.M.); 2024241023@bsu.edu.cn (W.X.); 2024241013@bsu.edu.cn (X.L.); 2 Beijing Key Laboratory of Sports Performance and Skill Assessment, Beijing Sport University, Beijing 100084, China; 3 Key Laboratory for Performance Training and Recovery of the General Administration of Sport of China, Beijing Sport University, Beijing 100084, China; 4 Department of Physical Education, Guangxi Medical University, Nanning 530021, China; wangyulong@sr.gxmu.edu.cn; 5 Public Physical Education Department, Linyi City Vocational College, Linyi 276000, China; 6 Faculty of Sports Science and Coaching, Sultan Idris Education University, Tanjong Malim 35900, Malaysia

**Keywords:** biofeedback, rehabilitation exercise, movement quality, wearable sensors, telerehabilitation

## Abstract

**Highlights:**

**What are the main findings?**

- Sensor-based rehabilitation exercise research has shifted from simple movement recording toward movement-quality assessment, feedback generation, and rehabilitation guidance.
- The 55 included studies showed wide use of inertial sensors, vision/depth cameras, surface electromyography, pressure/force sensors, and mobile or remote platforms, but substantial heterogeneity remained in metrics, algorithms, feedback strategies, and validation settings.

**What are the implications of the main findings?**

- Sensor-derived movement-quality metrics may help bridge the gap between prescribed rehabilitation exercise and actual exercise execution, particularly when rehabilitation exercise is performed outside direct therapist supervision.
- Future systems should move beyond technical accuracy validation toward real-world clinical validation, standardized task-specific movement-quality metrics, and feedback-enabled support for individualized rehabilitation progression.

**Abstract:**

**Background/Objectives:** Sensor-based movement assessment is increasingly used to quantify movement execution quality and support feedback-guided rehabilitation exercise, particularly in home-based and remote rehabilitation contexts. However, exercise adherence, movement execution quality, and rehabilitation progress remain difficult to monitor continuously and objectively outside direct therapist supervision. This scoping review aimed to map the current applications of sensor-based biofeedback and movement-quality assessment systems for rehabilitation exercise and to identify evidence gaps. **Methods:** This review followed established scoping review methodology and PRISMA-ScR guidance. PubMed/MEDLINE, Web of Science Core Collection, and IEEE Xplore were searched, and Google Scholar was used for supplementary searching. English-language studies published from January 2014 to May 2026 were eligible if they involved rehabilitation-related populations, sensor-based movement assessment, biofeedback, or training guidance. Data were charted and narratively synthesized according to rehabilitation context, sensor technology, movement-quality metrics, computational approaches, feedback strategies, real-time or remote functions, and reported outcomes. **Results:** Fifty-five studies published between 2015 and 2026 were included. The evidence covered neurological, musculoskeletal and orthopedic, balance and vestibular, fall-prevention, home-based, and telerehabilitation applications. Technologies included inertial sensors, smartphones, vision/depth cameras, surface electromyography, pressure/force sensors, and multisensor systems. Movement-quality metrics included range of motion, postural stability, gait characteristics, loading, muscle activation, movement correctness, repetition count, and task completion quality. Feedback was visual, auditory, vibrotactile, app-based, avatar-based, therapist-facing, or remote-platform-based. Most evidence came from feasibility, technical validation, algorithmic validation, and small-sample clinical studies. **Conclusions:** Sensor-based systems may help translate rehabilitation exercise performance into quantifiable and feedback-enabled information. Future research should strengthen real-world validation, standardize task-specific movement-quality metrics, and clarify how feedback mechanisms can support individualized rehabilitation progression.

## 1. Introduction

Rehabilitation is an essential component of healthcare systems, particularly for individuals with neurological, musculoskeletal, postoperative, balance-related, and age-related functional limitations. The World Health Organization has emphasized rehabilitation as a key component of universal health coverage, and global estimates suggest that approximately 2.4 billion people live with health conditions that may benefit from rehabilitation [1,2]. As rehabilitation demand increases, many patients are required to perform therapeutic exercises in home-based, community, or remote settings, where direct therapist supervision is limited [3,4]. In these contexts, the key challenge is not only whether patients complete the prescribed exercises, but also whether they perform them with sufficient quality, safety, and consistency.

Therapeutic exercise is commonly prescribed using parameters such as exercise type, frequency, intensity, duration, and progression. However, these prescription elements do not fully capture the quality of movement execution. Patients may complete the prescribed number of repetitions while showing insufficient range of motion (ROM), poor postural control, asymmetrical gait, inadequate limb loading, compensatory trunk movement, or inappropriate muscle activation. Such execution-related problems are particularly difficult to monitor during unsupervised rehabilitation and may reduce the intended training stimulus, delay functional recovery, or increase the risk of pain, falls, or reinjury [3,4]. Therefore, rehabilitation assessment increasingly needs to move beyond adherence and training volume alone toward objective evaluation of movement quality and task completion quality.

Sensor-based movement assessment provides a potential solution to this gap. Wearable inertial measurement units (IMUs), accelerometers, gyroscopes, smartwatches, surface electromyography (sEMG), pressure or force sensors, smartphone-based sensors, RGB-D cameras, and markerless pose-estimation systems can collect kinematic, kinetic, physiological, and electromyographic information during rehabilitation exercises [5,6,7,8,9,10]. The wider availability of smartphones, low-cost wearable devices, mobile applications, and cloud-based platforms has also made sensor-supported home-based and remote rehabilitation more feasible [5,9,10]. Recent work has further applied markerless motion capture, deep learning, real-time pose estimation, and on-device artificial intelligence (AI) to support rehabilitation exercise recognition, movement-error detection, and remote monitoring [11]. When sensor-derived data are translated into visual, auditory, vibrotactile, avatar-based, mobile-application, therapist-facing, or remote-platform feedback, these systems may support movement correction, motor learning, adherence monitoring, and individualized rehabilitation guidance [7,10].

Despite these developments, the evidence remains heterogeneous. Studies differ substantially in rehabilitation populations, exercise tasks, sensor configurations, sensor placement, reference standards, movement-quality metrics, computational models, feedback modalities, real-time capability, remote functions, and clinical outcome measures [5,6,7,8,9,10,11]. Some studies involve clinical interventions in patients, whereas others focus on feasibility testing, usability evaluation, technical validation, algorithm development, or healthy-participant rehabilitation-oriented validation. This heterogeneity makes it difficult to answer a single pooled effectiveness question across the entire field. Instead, it indicates the need for a scoping review that can map the conceptual boundaries, technical pathways, application contexts, and evidence gaps of sensor-based movement-quality assessment and biofeedback in rehabilitation exercise.

Previous reviews have provided important insights into biofeedback in rehabilitation, wearable movement sensors, inertial-sensor-based lower-limb exercise assessment, digital home rehabilitation, and telerehabilitation [5,6,7,8,9]. However, many existing reviews have been disease-specific, sensor-specific, feedback-specific, task-specific, or setting-specific. They have not fully mapped the complete pathway through which sensor-derived movement data are transformed into movement-quality constructs, computational approaches, feedback strategies, and rehabilitation guidance across rehabilitation exercise contexts. This pathway-level synthesis is important for home-based and remote rehabilitation because therapists require clinically interpretable information on how prescribed exercises are actually performed outside direct supervision, rather than only whether exercise sessions are completed.

Accordingly, this scoping review aimed to systematically map current applications of sensor-based movement-quality assessment and biofeedback systems for rehabilitation exercise. Specifically, the review examined rehabilitation populations and settings, exercise tasks, sensor technologies, movement-quality metrics, computational approaches, feedback strategies, real-time and remote functions, individualized or adaptive features, reported outcomes, and evidence gaps. By organizing the evidence along the pathway from sensor data acquisition to movement-quality assessment, feedback delivery, and rehabilitation guidance, this review seeks to clarify the current state of the field and identify priorities for future real-world validation, task-specific metric standardization, and feedback-enabled support for individualized rehabilitation progression.

## 2. Methods

### 2.1. Review Design

This study adopted a scoping review methodology to map the research distribution, technical characteristics, application contexts, and evidence gaps related to sensor-based biofeedback systems for rehabilitation exercise assessment and guidance. The design and reporting of this review were informed by the methodological framework for scoping studies proposed by Arksey and O’Malley, the methodological enhancements suggested by Levac et al., the JBI guidance for scoping reviews, and the PRISMA-ScR reporting guideline [12,13,14,15].

A scoping review design was considered appropriate because the evidence base included diverse rehabilitation populations, sensor technologies, exercise tasks, feedback modalities, computational approaches, implementation settings, and outcome measures. The aim was therefore to map the evidence landscape and identify research gaps rather than to estimate the pooled effectiveness of a single intervention. The included studies did not provide sufficiently homogeneous populations, interventions, comparators, outcomes, or study designs for meta-analysis.

Before conducting the review, the research team developed an internal review plan that specified the review questions, eligibility criteria, search strategy, study selection procedures, data-charting fields, and evidence synthesis approach. The protocol was not prospectively registered. To enhance transparency, it was retrospectively registered on the Open Science Framework on 27 June 2026 (OSF Registries; OSF Preregistration; registration identifier: w382t; OSF project/registration URL: https://osf.io/w382t (accessed on 3 August 2026)). No substantive deviations from the planned eligibility criteria, search strategy, data-charting domains, or synthesis approach occurred. A completed PRISMA-ScR checklist is provided in Supplementary Table S6.

### 2.2. Research Questions

This review was structured around the evidence pathway from sensor data acquisition to movement-quality assessment, feedback generation, and rehabilitation guidance. The review questions focused on the rehabilitation populations and contexts in which sensor-based biofeedback systems have been applied, the rehabilitation exercise tasks they support, the sensor technologies, movement-quality metrics, computational approaches, and feedback strategies used; whether these systems provide real-time feedback, remote supervision, individualized features, or adaptive functions; and what evidence gaps remain in relation to clinical validation, real-world application, long-term effectiveness evaluation, and metric standardization.

### 2.3. Eligibility Criteria

Eligibility criteria were defined using the Population–Concept–Context (PCC) framework [15]. The population included individuals requiring rehabilitation exercise, functional recovery, or rehabilitation-related exercise guidance, including but not limited to neurological, musculoskeletal or orthopedic, vestibular or balance-related, postoperative, chronic pain, fall-risk, and other rehabilitation populations. Studies involving healthy participants were also eligible when the task design or intended application was explicitly related to rehabilitation exercise assessment, movement-quality evaluation, biofeedback, telerehabilitation, therapeutic exercise guidance, or rehabilitation system development.

In this review, movement quality was operationally defined as sensor-derived information describing how a rehabilitation exercise or rehabilitation-related movement task was performed, rather than only whether it was completed. This included, where applicable, range of motion, joint angles, postural stability, trunk sway, gait symmetry, step width, foot progression angle, loading or weight distribution, muscle activation, compensatory movement, movement correctness, repetition validity, and task completion quality. Movement quality was therefore treated as a multidimensional and task-dependent construct rather than as a single universal clinical outcome.

The concept included sensor-based rehabilitation exercise assessment, movement-quality monitoring, biofeedback, remote monitoring, feedback generation, and rehabilitation guidance. Eligible sensor-based technologies were defined as wearable, mobile, vision-based, pressure/force, electromyographic, motion-capture, or multisensor systems that collected human kinematic, kinetic, physiological, or electromyographic data during rehabilitation exercise or rehabilitation-related movement tasks. Eligible technologies included inertial measurement units, accelerometers, gyroscopes, magnetometers, surface electromyography, pressure or force sensors, vision or depth cameras, marker-based or markerless motion capture, smartphone sensors, and multisensor systems. Systems based only on questionnaires, self-reported exercise logs, conventional clinical scales, or therapist observation without sensor-based data acquisition were excluded.

Assessment-only, algorithm-validation, model-development, and remote-monitoring studies were included when they were explicitly designed to support rehabilitation exercise assessment or future feedback generation. These studies were included as secondary evidence to characterize technical pathways and translational potential, but they were not considered equivalent to clinically validated biofeedback interventions.

The context included clinical rehabilitation, outpatient physical therapy, community rehabilitation, home-based rehabilitation, telerehabilitation, laboratory-simulated rehabilitation, and real-world rehabilitation-related settings. Eligible tasks included balance training, postural control, gait training, sit-to-stand tasks, lower- or upper-limb functional training, range-of-motion exercises, aquatic or swimming rehabilitation, fall-prevention exercises, and home-based therapeutic exercise.

Studies were excluded if they focused only on athletic performance, general fitness training, consumer exercise tracking, daily activity monitoring, disease diagnosis, risk prediction, fall detection, step counting, or general physical activity monitoring without rehabilitation exercise assessment, movement-quality evaluation, feedback guidance, or therapeutic exercise execution. Reviews, systematic reviews, scoping reviews, meta-analyses, protocols, editorials, commentaries, book chapters, patents, animal studies, in vitro studies, purely simulation-based studies, non-peer-reviewed materials, and studies without extractable information were also excluded.

This review included original research, including randomized controlled trials, non-randomized controlled studies, crossover studies, single-arm intervention studies, feasibility studies, usability studies, mixed-methods studies, system development and validation studies, sensor accuracy validation studies, algorithm or model validation studies, and conference abstracts with extractable information. The search covered studies published from January 2014 to May 2026 and was limited to English-language literature. The year 2014 was selected to provide a contemporary review window covering the rapid development and wider application of low-cost inertial sensors, smartphone-based sensing, markerless pose-estimation technologies, mobile health systems, and telerehabilitation platforms in rehabilitation exercise assessment and feedback. Although the search period started in 2014, the earliest eligible study identified after screening was published in 2015.

### 2.4. Information Sources and Search Strategy

A comprehensive literature search was conducted to identify studies on sensor-based rehabilitation exercise assessment, movement-quality monitoring, and biofeedback guidance. PubMed/MEDLINE, Web of Science Core Collection, and IEEE Xplore were searched. Google Scholar was used for supplementary searching. The search period was from January 2014 to May 2026, and only English-language publications were included.

For PubMed/MEDLINE and Web of Science Core Collection, two complementary search strategies were used. The first strategy primarily covered rehabilitation exercise assessment and movement-quality evaluation. The second strategy primarily covered rehabilitation exercise feedback, biofeedback, remote supervision, and rehabilitation guidance. Because IEEE Xplore has limited support for long and complex Boolean search strings, and because sensor-system, pose-estimation, movement-recognition, mobile-health, and remote-feedback studies are often published in the engineering and technical literature, a grouped search strategy was used for IEEE Xplore. These grouped searches covered inertial sensors, wearable sensors, surface electromyography, pose estimation, mobile applications, and remote rehabilitation feedback.

Google Scholar was used as a supplementary source to identify potentially relevant engineering, digital health, human–computer interaction, and conference literature that might not be fully captured by structured database searches. Eight highly relevant keyword combinations were used. For each keyword combination, the first 50 records ranked by relevance were screened. This was applied as a pragmatic supplementary-search strategy because Google Scholar returns relevance-ranked results and was used to identify additional highly relevant records rather than to replace structured database searches. A total of 216 Google Scholar records were retained for deduplication with database records. The complete search strategies are provided in Supplementary Table S2.

### 2.5. Study Screening and Eligibility Assessment

All retrieved records were first imported into EndNote for duplicate removal. After deduplication, two reviewers independently screened titles and abstracts to exclude clearly irrelevant studies. Full texts were then obtained for potentially eligible records and assessed against the predefined inclusion and exclusion criteria. Disagreements during screening were resolved through discussion; if consensus could not be reached, a third reviewer made the final decision.

During full-text screening, particular attention was paid to whether the study involved rehabilitation or rehabilitation-related exercise tasks, whether sensors were used to collect human movement or physiological signals, whether movement quality, movement performance, or training completion was analyzed, whether feedback, guidance, monitoring, or remote management functions were provided, and whether the study reported information relevant to the review questions. The study selection process is presented using a PRISMA-ScR flow diagram.

### 2.6. Data Charting Process

A predefined data-charting form was used for data extraction and was iteratively refined during pilot extraction according to the characteristics of the included literature. Extracted information included bibliographic information, study design, rehabilitation population and setting, sample characteristics, exercise task, intervention duration, sensor type and placement, input signals, reference standard, movement-quality metrics, algorithm or model, feedback modality, real-time capability, remote function, individualized features, clinical or functional outcomes, adherence, usability, safety, main findings, study limitations, and evidence category. The complete study-level extracted information is provided in Supplementary Table S1.

Data were initially extracted by one reviewer and cross-checked by two additional reviewers. For studies with incomplete or unclear information, judgments were based preferentially on the full-text methods, results, figures, tables, and Supplementary Materials. If information could still not be confirmed, it was marked as “not reported” or “unclear,” and no inferential imputation was performed.

### 2.7. Evidence Categorization and Data Synthesis

Given the substantial variation in study design and application maturity in this field, included studies were categorized by evidence type after data charting. According to study population, study objective, and application stage, studies were classified as clinical intervention studies, feasibility or usability studies, technical system validation studies, algorithm or model validation studies, healthy-participant simulated rehabilitation studies, and conference abstracts or early proof-of-concept studies. Studies were also labeled as core evidence or secondary evidence according to whether they directly involved real rehabilitation or disease populations, included rehabilitation exercise tasks, provided movement-quality assessment or feedback guidance, and reported clinical, functional, adherence, or usability outcomes. This classification was not used to exclude studies, but to help interpret evidence maturity and translational value.

A combination of descriptive statistics and thematic synthesis was used. First, frequencies and proportions were summarized for publication year, country or region, study design, rehabilitation population, rehabilitation setting, sensor type, feedback modality, real-time capability, and evidence category. Second, thematic synthesis was conducted around the rehabilitation application pathway, including sensor data acquisition, movement-quality assessment, computational or rule-based modeling, feedback generation, rehabilitation exercise guidance, and prescription-related functions. Third, to strengthen sensor-category-based synthesis, studies were summarized according to major sensor technology categories, including wearable inertial and smartphone-based motion sensors, vision/depth and markerless pose-estimation systems, pressure/force/load sensors, electromyography-based systems, and multisensor or mobile/cloud-supported systems. Because the included studies were highly heterogeneous in populations, rehabilitation tasks, sensor configurations, feedback strategies, computational approaches, and outcome measures, no meta-analysis was performed, and no pooled estimates of intervention effects were calculated.

### 2.8. Critical Appraisal

According to JBI guidance and PRISMA-ScR recommendations for scoping reviews, methodological quality appraisal is not a mandatory component of scoping reviews, particularly when the aim is to define the scope of evidence, map the research landscape, and identify research gaps [14,15]. Therefore, this review did not conduct a formal risk-of-bias assessment using a standardized appraisal tool, nor were studies excluded on the basis of methodological quality. However, in the synthesis and discussion, the maturity and limitations of the current evidence were interpreted with consideration of study design, sample size, inclusion of real patients, clinical validation, follow-up duration, and degree of real-world application.

## 3. Results

### 3.1. Study Selection

**Figure 1:**
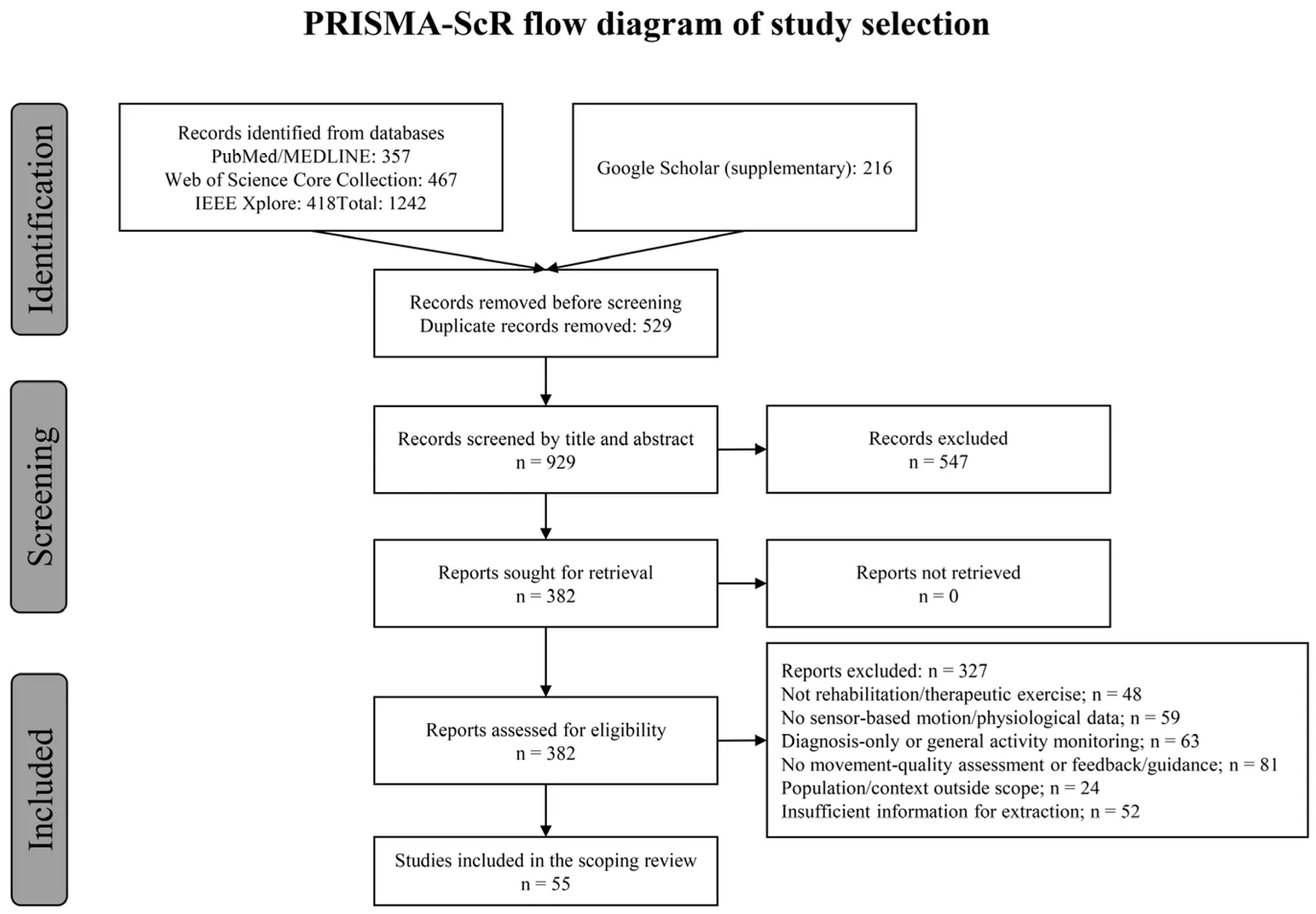
PRISMA-ScR flow diagram of study selection. The flow diagram shows the identification, screening, eligibility assessment, and inclusion process for studies included in this scoping review.

The database search identified 1242 records, including 357 from PubMed/MEDLINE, 467 from Web of Science Core Collection, and 418 from IEEE Xplore. An additional 216 records were identified through supplementary searching in Google Scholar. After duplicate removal, 929 records entered title and abstract screening. A total of 547 records were excluded at this stage because they were clearly inconsistent with the eligibility criteria, leaving 382 reports for full-text assessment. During full-text assessment, 327 reports were excluded for the following reasons: not involving rehabilitation training or therapeutic exercise tasks (n = 48), not using sensors to collect human movement or physiological signals (n = 59), focusing only on general physical activity monitoring or diagnosis-only monitoring (n = 63), not providing information related to movement-quality assessment or feedback guidance (n = 81), involving populations or application contexts outside the scope of this review (n = 24), or providing insufficient information for data extraction (n = 52). Finally, 55 studies were included in this scoping review. The study selection process is shown in Figure 1.

### 3.2. General Characteristics of Included Studies

**Figure 2:**
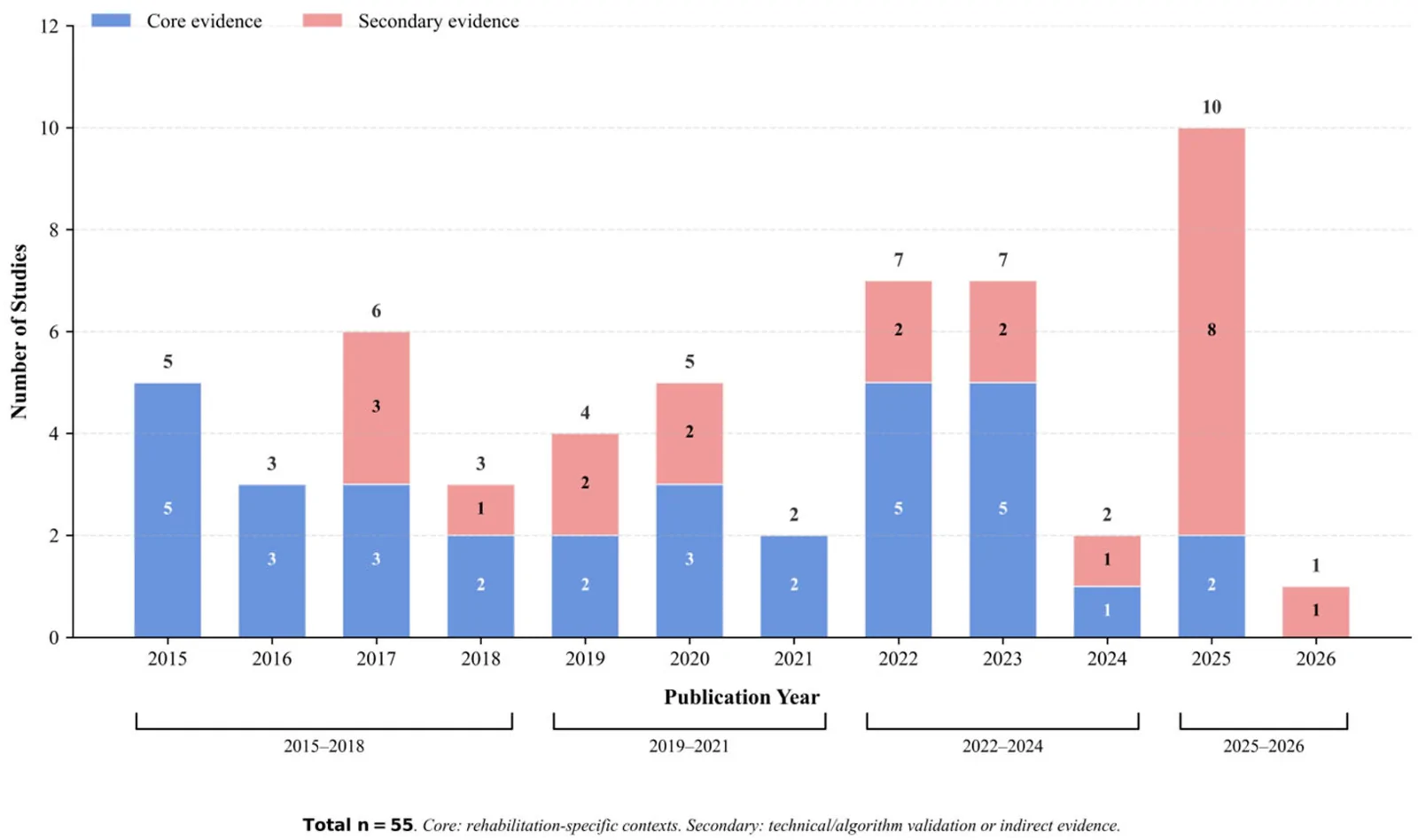
Annual distribution and evidence categories of included studies from 2015 to 2026. The stacked bar chart shows the number of included studies published each year, stratified by evidence category. Core evidence refers to studies directly involving rehabilitation patients, disease populations, or clearly defined rehabilitation contexts, whereas secondary evidence includes healthy-participant validation, technical or system validation, algorithm or model validation, conference abstracts, or indirect rehabilitation-support evidence.

The 55 included studies were published between 2015 and 2026. Among them, 38 studies were published in 2019 or later, and 27 were published in 2022 or later, suggesting a recent increase in research on sensor-based rehabilitation exercise assessment and feedback. The annual publication trend is shown in Figure 2.

The included studies were conducted across multiple regions, including Europe, Asia, North America, and Oceania. Study types included randomized or controlled intervention studies; non-randomized or crossover clinical studies; feasibility and usability studies; system or device validation studies; and algorithm, model, or dataset validation studies. According to study population, study objective, and application maturity, 33 studies were classified as core evidence. These studies mainly involved real rehabilitation patients, disease populations, postoperative rehabilitation patients, or clearly defined rehabilitation contexts. The remaining 22 studies were classified as secondary evidence, including healthy-participant validation, technical prototype evaluation, algorithm validation, dataset studies, and conference abstracts. The general characteristics of the included studies are presented in Table 1, and the complete study-level information is provided in Supplementary Table S1.

### 3.3. Rehabilitation Populations, Application Contexts, and Exercise Tasks

The included studies covered multiple rehabilitation domains, mainly including neurological rehabilitation, musculoskeletal and orthopedic rehabilitation, balance and vestibular rehabilitation, fall prevention in older adults, home-based rehabilitation, and telerehabilitation. Studies related to neurological rehabilitation involved populations with stroke, Parkinson’s disease, multiple sclerosis, cerebral palsy, cerebellar ataxia, mild cognitive impairment, and hemiparetic gait [16,17,18,19,20,21,22,23,24,25,26,27]. Musculoskeletal and orthopedic rehabilitation studies mainly involved total knee replacement rehabilitation, knee osteoarthritis, shoulder adhesive capsulitis, chronic ankle instability, low back pain, spinal posture control, and knee-extension rehabilitation [10,28,29,30,31,32,33,34,35,36,37,38,39,40]. Studies on balance, vestibular rehabilitation, and fall prevention mainly focused on multiple sclerosis, persistent postural-perceptual dizziness, chemotherapy-induced peripheral neuropathy, mild cognitive impairment, older adults at risk of falling, and healthy participants performing simulated balance tasks [17,19,25,27,41,42,43,44,45,46,47].

**Figure 3:**
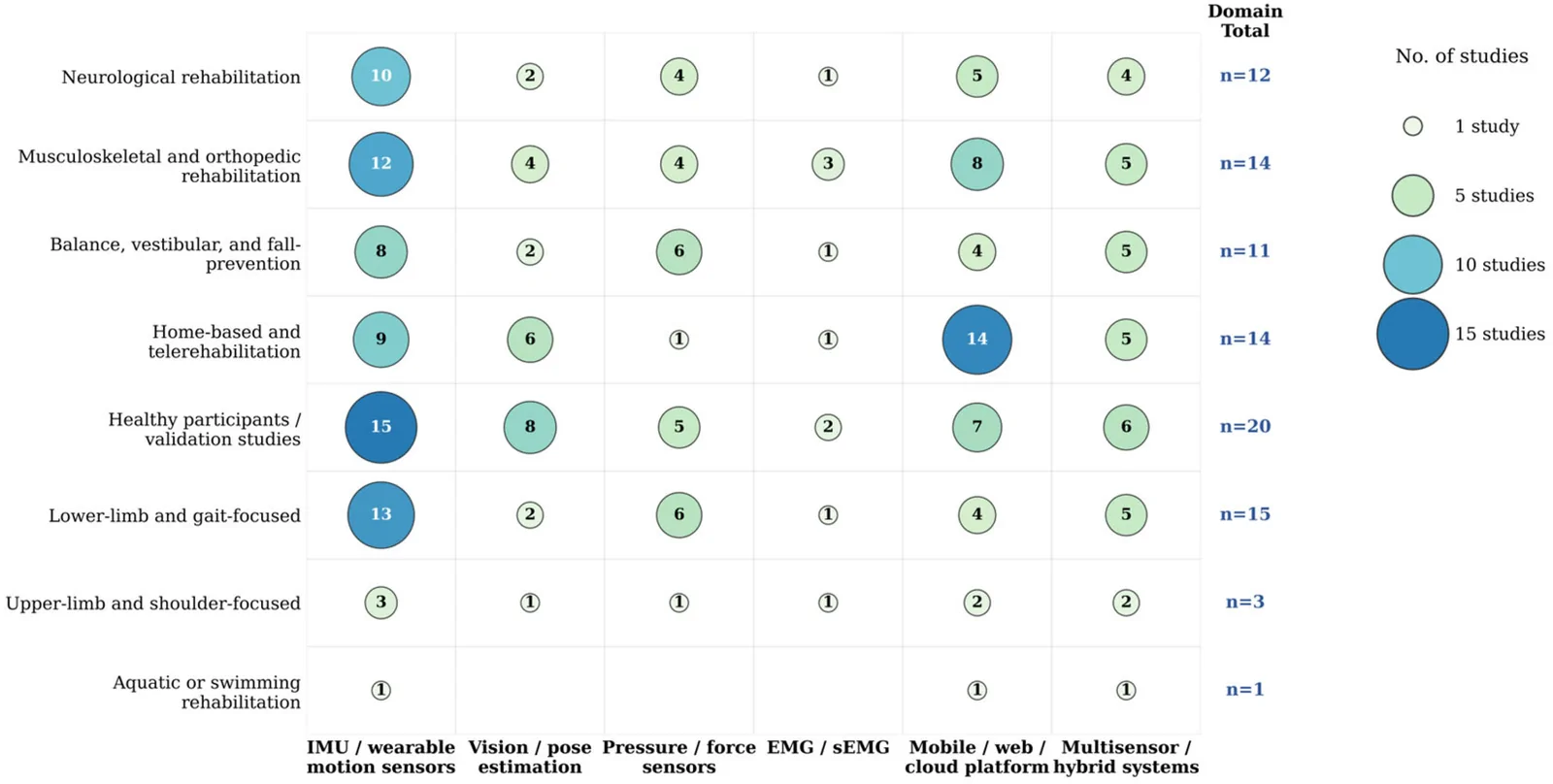
Evidence map linking rehabilitation domains and sensor technologies. The bubble plot maps rehabilitation/application domains against sensor or technology categories. Bubble size and color intensity indicate the number of studies mapped to each domain–technology combination. Categories are not mutually exclusive because a single study could involve more than one rehabilitation domain and more than one technology category.

The rehabilitation tasks were mainly concentrated on lower-limb and gait training, balance and postural control, upper-limb and shoulder–hand functional training, range-of-motion exercises, sit-to-stand tasks, weight-bearing training, and home-based therapeutic exercise. Some studies recruited healthy participants; however, their task designs or system objectives explicitly targeted rehabilitation exercise assessment, biofeedback, telerehabilitation, or rehabilitation system development. These studies were therefore included as secondary evidence [11,30,31,39,41,46,48,49,50,51,52,53,54,55,56,57,58,59,60,61]. The evidence distribution across rehabilitation populations and application contexts is shown in Figure 3, and the rehabilitation domains, exercise tasks, and representative studies are summarized in Table 2.

### 3.4. Sensor Technology Categories and Data Acquisition Pathways

Wearable inertial and smartphone-based motion sensors represented the predominant data-acquisition pathway. A total of 43 studies used or involved inertial measurement units (IMUs), accelerometers, gyroscopes, magnetometers, smartwatches, smartphone-based inertial sensors, or similar wearable motion-capture devices [10,17,18,19,20,21,22,23,25,26,27,28,30,31,32,33,34,35,36,37,38,39,40,41,43,44,45,46,47,48,54,56,57,58,59,60,61,62,63,64,65,66,67]. These systems were commonly used to estimate joint angles, range of motion (ROM), trunk sway, gait parameters, movement rhythm, repetition count, and training completion. Their frequent use reflects their portability, relatively low cost, suitability for real-time processing, and compatibility with home-based and remote rehabilitation settings.

Vision/depth and markerless pose-estimation systems formed the second major technology category. Eighteen studies used or involved RGB cameras, depth cameras, markerless motion capture, skeletal key points, pose-estimation models, or video datasets [11,26,29,31,37,49,50,51,53,54,55,56,58,61,63,64,65,68]. These systems were mainly used for non-contact movement assessment, rehabilitation exercise recognition, joint-angle estimation, movement-error classification, and remote monitoring. Compared with wearable sensors, vision-based systems can capture multijoint or whole-body movement without requiring multiple body-worn devices, although their performance may be affected by camera placement, occlusion, lighting conditions, privacy concerns, and differences between laboratory and home environments.

Pressure-, force-, and load-related sensors appeared in 15 studies [16,18,20,21,24,26,30,32,37,41,42,44,47,52,58]. These technologies were mainly used to quantify plantar pressure, limb loading, tibial load, weight distribution, center-of-pressure-related balance indicators, or haptic feedback parameters. They were particularly relevant for gait retraining, balance rehabilitation, partial weight-bearing training, fall-prevention exercises, and lower-limb rehabilitation tasks. Five studies involved electromyography or surface electromyography (sEMG) signals [28,30,34,62,68], mainly to assess muscle activation, exercise execution, or grasp-related function. In addition, 31 studies incorporated smartphones, mobile applications, web-based systems, cloud platforms, or remote data synchronization functions, indicating the increasing integration of sensor systems with mobile health and telerehabilitation platforms [10,11,18,22,23,25,30,31,33,35,36,39,40,41,42,48,49,50,51,52,53,54,58,59,60,61,62,64,65,67,68].

Table 3 summarizes the major sensor technology categories, movement-quality targets, feedback or application functions, and key translational considerations identified across the included studies. Categories are not mutually exclusive because a single study could involve more than one sensor type, feedback modality, or implementation platform.

### 3.5. Movement-Quality Constructs and Computational Approaches

Movement quality was not represented by a single universal metric across the included studies. Instead, it was operationalized through multiple kinematic, biomechanical, physiological, and behavioral indicators linked to rehabilitation goals. The most common constructs included movement correctness, movement-error classification, repetition validity, task completion, and training completion [10,11,22,29,35,36,39,40,48,49,50,51,53,54,55,59,60,63,64,65,67,68]. ROM and joint-angle metrics were frequently used in studies involving shoulder adhesive capsulitis, knee rehabilitation, knee-extension training, sit-to-stand tasks, spinal posture control, and upper-limb functional training [26,30,31,34,35,36,38,39,40,50,54,56,64,68].

Balance, trunk sway, and postural stability metrics were mainly reported in balance, vestibular, and fall-prevention rehabilitation studies [17,19,20,25,27,41,42,43,44,45,46,47,58]. Gait-quality metrics included step width, foot progression angle, gait speed, gait cycle, gait symmetry, plantar pressure, tibial loading, and weight distribution [16,18,20,21,23,26,32,37,52,57]. Muscle activation and force-related indicators were less frequently reported and mainly involved sEMG, force myography, local loading, plantar force, or quadriceps activation [24,30,34,62]. Overall, movement-quality assessment has expanded from isolated parameters such as ROM, repetition count, or gait speed toward multidimensional constructs involving movement correctness, compensatory patterns, postural strategy, loading control, task completion quality, and exercise adherence.

Computational approaches could be grouped into three broad categories. The first included rule-based, threshold-based, and conventional signal-processing methods, which were commonly used for real-time feedback, repetition recognition, target-angle detection, postural-deviation detection, and loading feedback [19,20,25,26,32,34,35,36,38,39,40,41,42,43,45,46,47,52,57,61,62,64]. The second included machine learning and deep learning methods, which were mainly used for movement recognition, movement segmentation, error classification, pose-based assessment, and movement-quality scoring [11,22,29,48,49,50,51,53,54,55,59,60,63,65,67,68]. The third included individualized calibration and biomechanical modeling approaches, which generated feedback rules based on baseline performance, target joint angles, individualized gait parameters, patient-specific thresholds, or therapist-confirmed correct movements [17,18,19,20,25,32,35,36,37,40,45,64]. These computational approaches suggest a gradual transition from simple signal detection toward automated and individualized movement-quality interpretation, although many algorithmic studies remained at the technical validation stage.

### 3.6. Feedback Strategies, Real-Time/Remote Features, and Reported Outcomes

Visual, mobile-application, and virtual-interface feedback were the most common feedback modalities. Thirty-four studies involved feedback delivered through screens, mobile applications, virtual avatars, gamified interfaces, skeleton overlays, color cues, movement trajectories, or progress charts [10,11,18,20,22,23,25,27,28,29,30,31,33,34,35,36,37,39,40,43,49,51,52,54,55,58,59,60,62,63,64,65,67,68]. These feedback formats were mainly used to present target attainment, movement correctness, ROM, postural deviation, exercise completion, or progress information. Auditory or sonification feedback appeared in 12 studies and was mainly used for gait, balance, trunk control, or dynamic movement tasks [20,21,27,28,30,32,37,38,43,46,57,65]. Vibrotactile or haptic feedback appeared in nine studies and was mainly used for balance control, trunk sway, gait-parameter adjustment, posture correction, or sensory augmentation [16,17,19,25,28,41,42,45,52].

Therapist-facing and remote-management functions were also common. Thirty-six studies involved therapist review, clinician monitoring, remote platforms, web portals, cloud synchronization, clinician-facing applications, or rehabilitation management interfaces [11,18,20,21,22,23,24,25,26,28,29,31,32,33,34,35,36,39,40,44,47,48,49,50,51,53,54,56,58,59,60,61,63,64,66,68]. These systems were used to record training completion, movement performance, ROM changes, adherence, symptom records, or rehabilitation progress, thereby supporting asynchronous supervision and therapist-led decision-making. However, most systems still relied on predefined thresholds, therapist-defined targets, or manual interpretation. Few studies demonstrated fully adaptive systems capable of automatically adjusting rehabilitation goals, feedback thresholds, exercise difficulty, or prescription parameters based on longitudinal patient performance.

**Figure 4:**
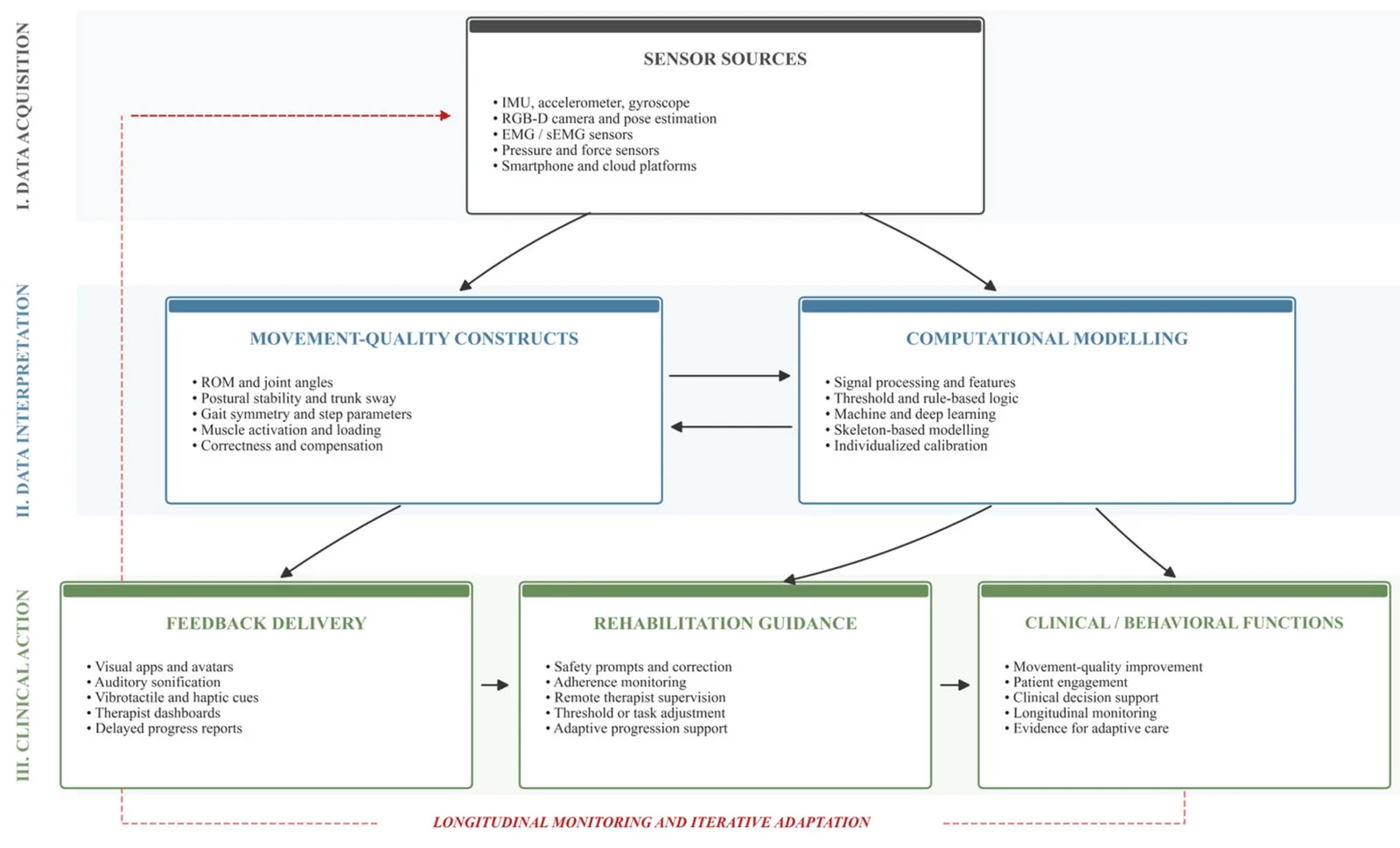
Conceptual framework for movement-quality feedback and rehabilitation prescription support. The framework illustrates how sensor-derived data can be translated into biomechanical constructs, computational models, feedback delivery, rehabilitation guidance, and expected clinical or behavioral functions. Solid arrows indicate the current assessment and feedback pathway, whereas the dashed loop represents longitudinal monitoring and the emerging transition toward adaptive rehabilitation prescription support.

Regarding real-time capability, 37 studies explicitly supported real-time or near-real-time feedback, processing, or display [10,16,17,18,19,20,21,22,25,26,27,28,32,33,34,35,36,37,38,39,40,41,42,43,44,45,46,47,51,52,53,57,61,62,64,65,66]. Four studies involved partly real-time, semi-online, or unclear real-time implementation [11,23,30,58]. Fourteen studies primarily used offline analysis, post-processing, or did not validate real-time feedback deployment [24,29,31,48,49,50,54,55,56,59,60,63,67,68]. Reported clinical and functional outcomes in patient-based studies included balance ability, gait performance, ROM, pain, functional questionnaires, quality of life, fall-related indicators, exercise adherence, usability, and patient experience. Some randomized controlled or intervention studies reported improvements in balance, gait, ROM, exercise adherence, or patient experience [17,18,19,20,27,28,35,36,37,40,43,45]. However, because the included studies differed substantially in populations, intervention durations, feedback modalities, implementation contexts, and outcome measures, this review did not pool effect sizes or draw definitive conclusions regarding the effectiveness of any specific intervention. Feedback strategies, implementation features, and reported outcomes are summarized in Supplementary Table S5. The overall pathway from sensor-derived data to movement-quality feedback and rehabilitation guidance is summarized in Figure 4.

## 4. Discussion

### 4.1. Principal Findings

This scoping review mapped 55 studies published between 2015 and 2026 on sensor-based movement-quality assessment and biofeedback for rehabilitation exercise. The findings indicate that this field is moving beyond simple movement recording toward systems that can quantify exercise execution quality, deliver feedback, support therapist monitoring, and potentially inform individualized rehabilitation progression. The evidence covered neurological, musculoskeletal and orthopedic, balance and vestibular, fall-prevention, home-based, and telerehabilitation contexts [10,16,17,18,19,20,21,22,23,24,25,26,27,35,36,37,38,39,40,43,45]. However, the current evidence remains distributed across different stages of maturity, including clinical intervention studies, feasibility and usability studies, technical validation, algorithm development, and healthy-participant rehabilitation-oriented validation [10,11,22,29,30,31,39,48,49,50,51,52,53,54,55,56,57,58,59,60,61,63,64,65,66,67,68].

The central implication of this review is that sensor-based systems may help address a persistent gap in rehabilitation exercise: the difference between prescribed exercise and actual exercise execution. In home-based and remote rehabilitation, therapists often know what exercises were prescribed but have limited objective information on how they were performed [4,5,9]. Sensor-derived metrics can make this process more visible by quantifying range of motion (ROM), postural stability, gait characteristics, loading, muscle activation, compensatory movement, repetition validity, and task completion quality [16,17,18,19,20,21,26,32,34,35,36,37,38,39,40,41,42,43,44,45,46,47,52,57,62]. This shift is clinically important because rehabilitation outcomes depend not only on adherence and training volume, but also on whether patients perform movements safely, accurately, and progressively [3,4,7].

At the same time, this review does not support a single conclusion about the effectiveness of sensor-based biofeedback as a uniform intervention. The included studies differed substantially in populations, sensor configurations, exercise tasks, feedback modalities, intervention duration, implementation setting, and outcome measures [10,17,18,19,20,35,36,37,40,43,45]. Therefore, the field is currently better understood as a heterogeneous and rapidly developing evidence landscape rather than as a mature intervention area suitable for one pooled effectiveness estimate. This interpretation is consistent with scoping review methodology, which is designed to map evidence types, clarify concepts, and identify research gaps in fields with substantial methodological and conceptual diversity [12,13,14,15].

### 4.2. Sensor Technology Pathways and Translational Challenges

Wearable inertial and smartphone-based motion sensors were the most common technology pathway. Their predominance is understandable because inertial measurement units (IMUs), accelerometers, gyroscopes, smartwatches, and smartphone sensors are portable, relatively inexpensive, suitable for repeated or continuous measurement, and compatible with home-based rehabilitation [6,8,10,18,20,25,32,35,36,39,40,41,56,62,64]. These systems have been used to monitor joint angles, ROM, trunk sway, gait parameters, movement rhythm, repetition count, and exercise completion [10,17,18,19,20,21,22,23,25,26,27,28,30,31,32,33,34,35,36,37,38,39,40,41,43,44,45,46,47,48,54,56,57]. However, clinical translation still depends on reliable sensor placement, calibration, patient adherence, signal quality, battery life, and the ability to present data in a clinically interpretable form [6,8,10,33,64].

Vision/depth cameras and markerless pose-estimation systems represented another major pathway. Their appeal lies in their ability to capture multijoint or whole-body movement without requiring patients to wear multiple devices, which is particularly relevant for remote physiotherapy, home-based exercise assessment, and automated movement-error classification [11,29,31,49,50,51,53,54,55,63,65,68]. Recent studies have used skeleton landmarks, markerless motion capture, deep learning, pose estimation, and on-device AI to support rehabilitation exercise assessment and remote monitoring [11,48,49,50,51,53,55,65,68]. However, performance in controlled laboratory environments may not transfer directly to rehabilitation patients or home settings, where lighting, camera angle, occlusion, assistive devices, slow movement speed, compensatory strategies, privacy concerns, and limited space may affect system accuracy and usability [31,50,51,53,58,63,68].

Pressure, force, load, and plantar-pressure sensors provided information that is difficult to obtain from kinematic sensors alone. These systems were especially relevant for gait retraining, partial weight-bearing training, balance rehabilitation, and fall-prevention exercises because they can quantify loading, weight distribution, plantar pressure, tibial load, or center-of-pressure-related indicators [16,18,20,21,24,26,32,37,41,42,44,47,52,58]. Surface electromyography (sEMG) and related muscle-activation sensing further expand assessment by capturing neuromuscular effort rather than movement alone [28,30,34,62,68]. Nevertheless, these systems often require more careful setup, calibration, and interpretation, which may limit scalability outside supervised clinical or laboratory environments [24,30,34,62].

Across these technology pathways, the main translational challenge is not simply whether a sensor can measure movement accurately. A technically accurate system must still provide information that patients can understand, therapists can interpret, and rehabilitation workflows can use [10,22,33,35,36,40,64]. For clinical implementation, the most useful system may not be the one with the most sensors or the most complex algorithm, but the one that provides reliable, interpretable, actionable, and low-burden information for rehabilitation decision-making [6,8,10,33].

### 4.3. Movement-Quality Constructs and Feedback Mechanisms

A key finding of this review is that movement quality was treated as a multidimensional and task-dependent construct. In some contexts, movement quality referred mainly to ROM or joint-angle attainment; in others, it reflected gait symmetry, trunk sway, load distribution, postural stability, muscle activation, movement correctness, or repetition validity [16,17,18,19,20,21,26,30,32,34,35,36,37,38,39,40,41,42,43,44,45,46,47,52,57,62,68]. This variability reflects the diversity of rehabilitation goals, but it also limits comparability across studies. Future research should therefore define movement-quality metrics according to specific rehabilitation tasks and clinical objectives rather than only according to what a given sensor can measure.

For example, movement quality in shoulder adhesive capsulitis rehabilitation may prioritize ROM, pain-limited movement, and compensation control [40]. Post-total knee arthroplasty or knee osteoarthritis rehabilitation may emphasize knee flexion and extension angles, quadriceps activation, training completion, and safe progression [35,36]. Balance and vestibular rehabilitation may focus on trunk sway, center-of-pressure-related indicators, postural stability, and sensory-condition-specific control [17,19,25,27,41,42,43,44,45,46,47,58]. Gait rehabilitation may require metrics such as step width, foot progression angle, gait speed, symmetry, plantar pressure, and loading [16,18,20,21,26,32,37,52,57]. These task-specific differences suggest that standardized movement-quality frameworks are needed before sensor-derived data can be consistently used for clinical decision-making.

Feedback is the mechanism through which sensor systems move from measurement to rehabilitation guidance. Visual, app-based, avatar-based, auditory, vibrotactile, haptic, therapist-facing, and remote-platform feedback can help patients recognize movement errors, adjust performance, and understand progress [10,16,17,18,19,20,21,22,25,27,28,30,32,35,36,37,39,40,41,42,43,45,46,52,54,57,62,65]. However, more feedback is not necessarily better. Motor-learning research indicates that feedback frequency, timing, content, and attentional focus can influence learning, retention, and transfer [69,70,71]. In the included studies, only a limited number of systems used individualized thresholds, faded feedback, or therapist-adjusted feedback strategies [18,20,25,35,36,40,64]. Future studies should clarify what feedback should be provided, when it should be provided, how frequently it should be delivered, and how feedback should be faded or individualized over time.

### 4.4. Home-Based Rehabilitation, Remote Supervision, and Adaptive Progression

Home-based and remote rehabilitation are particularly important application contexts for sensor-based movement-quality assessment. As rehabilitation demand increases, many patients must perform therapeutic exercise outside hospitals or rehabilitation centers [1,2,5,9,72]. Traditional home exercise programs often rely on paper instructions, videos, or self-reported logs, which provide limited information about adherence, movement execution quality, symptom response, and progression readiness [3,4]. Sensor-based systems can help convert home exercise performance into objective information that supports both patient self-regulation and therapist-led remote supervision [10,11,22,25,35,36,39,40,50,53,54,62,63,64].

On the patient side, real-time or near-real-time feedback can support target attainment, error correction, movement awareness, and engagement [10,18,20,25,35,36,39,40,62,65]. On the therapist side, remote platforms can summarize training completion, ROM changes, movement-quality metrics, adherence patterns, and symptom records [22,33,35,36,40,53,63,64]. This information may support asynchronous review and more timely adjustment of rehabilitation plans. However, remote supervision should not be equated with automated rehabilitation. Most existing systems still depend on therapist-defined targets, predefined thresholds, manual interpretation, or periodic prescription adjustment [10,22,33,35,36,40,64].

The next stage of development should therefore focus on adaptive progression. A clinically useful system should not only detect whether an exercise was completed, but also determine whether the movement was performed with sufficient quality, whether the patient is ready to progress, whether feedback thresholds should be adjusted, and whether therapist intervention is required. This requires integrating sensor-derived movement-quality metrics with symptom changes, patient-reported outcomes, adherence records, and therapist-defined clinical rules [10,22,35,36,40,53,63,64]. Such integration could help sensor-based systems move from exercise-monitoring tools toward individualized rehabilitation prescription support platforms.

### 4.5. AI-Enabled Future Directions

Recent advances in artificial intelligence (AI) may further expand the role of sensor-based rehabilitation assessment and feedback. In the included evidence, machine learning, deep learning, skeleton-based models, pose estimation, and on-device AI have already been used for rehabilitation exercise recognition, movement segmentation, error classification, movement-quality scoring, and remote monitoring [21,28,47,48,49,50,51,53,54,55,59,60,63,65,67,68]. These approaches may improve automated detection of exercise performance, but their clinical value depends on whether model outputs can be translated into understandable and actionable feedback for patients and therapists.

Vision transformers, foundation models, and large language models (LLMs) may become relevant to future rehabilitation systems by improving movement representation learning, multimodal data integration, patient-facing explanations, therapist-facing summaries, adherence support, and decision support. However, this remains an emerging direction rather than a mature clinical application in the included literature. Therefore, future research should be cautious not to equate AI model performance with clinical effectiveness. Algorithmic performance metrics such as accuracy, F1 score, or angular error are not sufficient on their own [11,29,48,49,50,51,53,54,55,59,60,63,65,67,68]. Future studies should evaluate whether AI-generated outputs are clinically meaningful, interpretable, safe, and useful for patients and therapists.

Particular attention should be paid to external validation across devices, rehabilitation populations, home environments, and abnormal movement patterns. AI systems should also be designed to support therapist-led care rather than replace clinical judgment. For rehabilitation applications, the key question is not only whether AI can classify movement correctly, but whether it can generate feedback and summaries that improve movement quality, adherence, safety, and rehabilitation progression [6,8,10,22,33,35,36,40,64].

### 4.6. Research Gaps and Future Priorities

Several priorities emerge from this review. First, more studies are needed in real rehabilitation patients and real-world home or community environments. Healthy-participant and laboratory validation studies are useful for early development, but they do not fully represent the pain, fatigue, cognitive limitations, compensatory movements, assistive-device use, and environmental variability observed in rehabilitation populations [11,29,30,31,39,41,46,47,48,49,50,51,52,53,54,55,56,57,58,59,60,61,66,67]. Second, task-specific movement-quality metrics require further standardization. Without clearer definitions of what constitutes acceptable or high-quality movement for specific rehabilitation tasks, it will remain difficult to compare systems, interpret feedback, or link sensor-derived metrics to clinical outcomes [16,17,18,19,20,21,26,30,32,34,35,36,37,38,39,40,41,42,43,44,45,46,47,52,57,62,68]. Third, feedback mechanisms need more systematic evaluation. Future studies should compare feedback modality, timing, frequency, complexity, and fading strategies, and should investigate whether these features improve motor learning, adherence, confidence, safety, and functional recovery [7,69,70,71]. Fourth, algorithmic validation should be linked more closely to clinical validation. Future research should move beyond technical performance and examine whether sensor-derived and AI-derived outputs are understandable to patients, useful to therapists, feasible in clinical workflows, and associated with meaningful rehabilitation outcomes [11,22,29,48,49,50,51,53,54,55,59,60,63,65,67,68]. Fifth, adaptive rehabilitation prescription remains at an early stage. Few systems currently demonstrate closed-loop progression based on longitudinal performance. Future systems should integrate movement-quality metrics, adherence, symptoms, patient-reported outcomes, and therapist-defined rules to support interpretable and clinically supervised progression decisions [10,22,35,36,40,53,63,64].

### 4.7. Limitations

This review also has limitations. First, only English-language publications were included, which may have led to the omission of relevant studies published in other languages. Second, the main databases searched were PubMed/MEDLINE, Web of Science Core Collection, and IEEE Xplore. Although Google Scholar was used as a supplementary source, some engineering conference papers, commercial system evaluations, grey literature, or studies indexed in other databases may have been missed. In addition, only the first 50 relevance-ranked Google Scholar records for each keyword combination were screened, which may have missed lower-ranked records. Third, because the aim of this scoping review was evidence mapping rather than effect synthesis, no formal risk-of-bias assessment or meta-analysis was performed, and the review cannot draw definitive conclusions regarding the effectiveness of any specific sensor-based feedback intervention. Fourth, the included studies were highly heterogeneous in populations, tasks, sensors, algorithms, feedback modalities, implementation settings, and outcome measures. Some classifications therefore required interpretation by the research team according to study objectives and system functions. Fifth, several included studies were conference abstracts, technical prototypes, or early validation studies with limited information completeness, and their findings should be interpreted cautiously as secondary evidence.

## 5. Conclusions

This scoping review mapped current evidence on sensor-based movement-quality assessment and biofeedback systems for rehabilitation exercise, with particular attention to home-based and remote rehabilitation. Across the 55 included studies, sensor-based systems were increasingly used to quantify how rehabilitation exercises were performed, including range of motion, joint angles, postural stability, gait characteristics, loading, muscle activation, compensatory movement, movement correctness, repetition validity, and task completion quality. These systems may help make rehabilitation exercise more objective, visible, and feedback-enabled, especially when patients train outside direct therapist supervision.

However, the current evidence remains heterogeneous and uneven in maturity. Many studies were feasibility studies, technical validation studies, algorithmic studies, healthy-participant validation studies, or small-sample clinical studies. Movement-quality metrics are not yet standardized, feedback mechanisms remain insufficiently understood, and long-term validation in real patients, real home environments, and routine rehabilitation workflows remains limited. Therefore, this review cannot determine the effectiveness of any specific sensor-based feedback intervention. Future research should develop task-specific and clinically interpretable movement-quality metrics, strengthen real-world validation, compare feedback strategies, and link sensor-derived or AI-derived outputs with meaningful clinical outcomes. More focused systematic reviews or meta-analyses will be needed when evidence becomes sufficiently homogeneous for specific populations, sensor technologies, feedback modalities, and outcomes.

## Figures and Tables

**Table 1:** General characteristics of the included studies (n = 55).

Characteristic	Category	n (%)
Publication period	2015–2018	17 (30.9)
	2019–2021	11 (20.0)
	2022–2024	16 (29.1)
	2025–2026	11 (20.0)
Geographical region	Europe	23 (41.8)
	Asia	17 (30.9)
	North America	12 (21.8)
	Oceania	3 (5.5)
Primary study design or application type	Randomized or controlled intervention study	9 (16.4)
	Non-randomized, single-arm, crossover, or comparative clinical intervention study	10 (18.2)
	Feasibility, usability, qualitative, or user-centred evaluation study	5 (9.1)
	Technical, device, or system validation study	18 (32.7)
	Algorithm, model, or dataset validation study	13 (23.6)
Evidence category	Core evidence	33 (60.0)
	Secondary evidence	22 (40.0)
Research or application setting	Laboratory or simulated validation setting	33 (60.0)
	Home-based, remote, or telerehabilitation setting	30 (54.5)
	Clinical, hospital, rehabilitation, or physiotherapy setting	17 (30.9)
	Community or real-world setting	4 (7.3)
Real-time capability	Real-time or near-real-time feedback, processing, or display	37 (67.3)
	Partly real-time or semi-online implementation	4 (7.3)
	Offline analysis, post-processing, or real-time capability not reported	14 (25.5)

**Table 2:** Rehabilitation populations, application contexts, and exercise tasks addressed in the included studies.

Rehabilitation/Application Domain	Conditions or Populations	Typical Settings	Representative Exercise Tasks	No. of Studies	Representative Studies
Neurological rehabilitation	Stroke, Parkinson’s disease, multiple sclerosis, cerebral palsy, hereditary cerebellar ataxia, mild cognitive impairment, hemiparetic gait	Clinical rehabilitation gym, laboratory, home-based rehabilitation, remote monitoring	Balance training, gait training, weight shifting, postural control, upper-limb functional training, grasp monitoring, smartwatch-supported home exercise, gait sonification	12	[16,17,18,19,20,21,22,23,24,25,26,27]
Musculoskeletal and orthopedic rehabilitation	Total knee replacement, knee osteoarthritis, shoulder adhesive capsulitis, low back pain, chronic ankle instability, knee extension rehabilitation, spinal posture control	Hospital or outpatient rehabilitation, home-based exercise, laboratory validation, physiotherapy clinic	Knee flexion/extension, short arc quadriceps exercise, sit-to-stand, shoulder ROM exercise, wall-walking stretch, cane stretch, squat, lunge, gait retraining, lumbar posture correction	14	[10,28,29,30,31,32,33,34,35,36,37,38,39,40]
Balance, vestibular, and fall-prevention rehabilitation	Older adults, fall-risk populations, multiple sclerosis, persistent postural–perceptual dizziness, chemotherapy-induced peripheral neuropathy, mild cognitive impairment, healthy participants in simulated balance tasks	Clinical physiotherapy, laboratory balance testing, community or older-adult settings, home or semi-supervised training	Static and dynamic balance tasks, upright stance, foam-surface standing, tandem stance, trunk sway control, weight shifting, obstacle walking, fall-prevention exercises	11	[17,19,25,27,41,42,43,44,45,46,47]
Home-based and telerehabilitation applications	Stroke, shoulder adhesive capsulitis, orthopedic rehabilitation, knee rehabilitation, general home exercise users, older adults, remote rehabilitation users	Home, remote rehabilitation platforms, mobile applications, cloud-based systems, therapist portals	Home exercise monitoring, remote therapist supervision, exercise completion tracking, ROM monitoring, mobile-app-guided exercise, virtual avatar feedback, asynchronous feedback	14	[10,11,22,25,35,36,39,40,50,53,54,62,63,64]
Healthy participants in rehabilitation-oriented validation studies	Healthy adults, healthy older adults, or healthy volunteers recruited for system, algorithm, or feasibility validation with explicit rehabilitation relevance	Laboratory, simulated rehabilitation setting, controlled home-like environment, motion analysis laboratory	Rehabilitation exercise recognition, movement-quality classification, squat/lunge assessment, knee-extension testing, gait retraining, posture feedback, sensor accuracy validation	20	[11,30,31,39,41,46,48,49,50,51,52,53,54,55,56,57,58,59,60,61]
Aquatic or swimming rehabilitation	Swimming rehabilitation users or simulated rehabilitation swimmers; primarily technical/case-study evidence	Swimming pool, aquatic rehabilitation or sport-therapy setting	Stroke-style recognition, stroke count, stroke period, trunk rotation symmetry, swimming intensity and fatigue-related parameters	1	[61]
Upper-limb and shoulder-focused rehabilitation	Stroke upper-limb impairment, shoulder adhesive capsulitis, hand/grasp dysfunction, upper-limb rehabilitation users	Home-based rehabilitation, clinical rehabilitation, mobile-app or wearable-sensor setting	Shoulder flexion, abduction, extension, internal/external rotation, wall-walking exercise, cane-assisted stretching, grasp monitoring, upper-limb home exercise	3	[22,24,40]
Lower-limb and gait-focused rehabilitation	Knee rehabilitation, knee osteoarthritis, knee replacement, chronic ankle instability, stroke gait impairment, Parkinson’s disease, healthy gait-retraining participants	Clinical rehabilitation, laboratory gait analysis, treadmill setting, home-based knee rehabilitation	Knee ROM training, sit-to-stand, short arc quadriceps exercise, partial weight-bearing gait, step width and foot progression angle retraining, gait speed and symmetry training	15	[16,18,20,21,23,26,30,32,34,35,36,37,39,52,57]

Notes. Categories are not mutually exclusive because a single study could involve more than one application context or task category, such as neurological rehabilitation with balance training or home-based orthopedic rehabilitation with mobile-app feedback. The “No. of studies” column therefore represents the number of studies mapped to each domain rather than mutually exclusive totals. Representative study identifiers correspond to the included-study numbering used in the Results section and Supplementary Table S1. ROM, range of motion.

**Table 3:** Sensor technology categories, movement-quality targets, feedback functions, and translational considerations.

Sensor or Technology Category	Typical Rehabilitation Tasks	Main Movement-Quality Targets	Feedback or Application Functions	Key Translational Considerations
Wearable inertial and smartphone-based motion sensors (n = 43)	Gait training, balance training, ROM exercises, sit-to-stand, squats, lunges, home exercise monitoring	Joint angles, ROM, trunk sway, gait parameters, movement rhythm, repetition count, task completion	Real-time or near-real-time feedback, mobile-app feedback, movement correction, repetition validation, remote monitoring	Portable and low-cost, but affected by sensor placement, calibration, signal drift, device adherence, and patient usability
Vision/depth cameras and markerless pose-estimation systems (n = 18)	Exercise recognition, posture assessment, low back or lower-limb exercise assessment, remote physiotherapy, home-based monitoring	Skeletal key points, joint angles, movement trajectory, movement correctness, error classification, task quality	Non-contact movement assessment, visual feedback, skeleton overlays, AI-supported exercise assessment, remote supervision	Reduces device-wearing burden, but may be affected by camera position, lighting, occlusion, privacy, and generalizability to patients with abnormal movement patterns
Pressure, force, load, and plantar-pressure sensors (n = 15)	Partial weight-bearing gait, balance rehabilitation, fall-prevention training, gait retraining, lower-limb loading tasks	Limb loading, plantar pressure, tibial load, weight distribution, center-of-pressure-related indicators	Loading feedback, haptic feedback, balance feedback, gait-parameter retraining, therapist monitoring	Clinically relevant for load management, but may require specialized hardware, calibration, and controlled measurement conditions
EMG/sEMG and muscle-activation sensing (n = 5)	Knee-extension rehabilitation, posture-related tasks, grasp monitoring, therapeutic exercise assessment	Muscle activation, quadriceps activation, grasp-related signals, movement effort	Muscle-activation feedback, exercise validation, therapist-facing monitoring	Provides physiological information not captured by kinematics alone, but is sensitive to electrode placement, signal noise, skin preparation, and interpretation burden
Multisensor, mobile, web-based, cloud, or remote-platform systems (n = 31)	Home-based rehabilitation, telerehabilitation, remote therapist supervision, adherence monitoring, multimodal rehabilitation assessment	Training completion, movement performance, adherence, ROM change, symptom records, task quality	Remote monitoring, asynchronous therapist review, app-guided feedback, cloud synchronization, rehabilitation management	Supports service continuity, but requires workflow integration, data prioritization, privacy protection, and clinically interpretable summaries

## Data Availability

No new data were created or analyzed in this study. Data sharing is not applicable to this article.

## Supplementary Materials

The following supporting information can be downloaded at https://www.mdpi.com/article/10.3390/healthcare14162450/s1, Table S1: Characteristics of all included studies; Table S2: Search strategies used for the scoping review; Table S3: Sensor technologies, placements, input signals, and reference standards used in the included studies; Table S4: Movement-quality constructs and computational approaches used in the included studies; Table S5: Feedback strategies, implementation features, and reported outcomes in the included studies; Table S6: PRISMA-ScR checklist.
