# Supplementary material for: Sensor-Based Movement Quality Assessment and Biofeedback for Rehabilitation Exercise: A Scoping Review with Implications for Home-Based and Remote Rehabilitation
Source: Healthcare (Basel). 2026 Aug 7;14(16):2450. doi: 10.3390/healthcare14162450 (PMC13512171; doi:10.3390/healthcare14162450)
Supplement: Supplementary file 1 [file healthcare-14-02450-s001.zip › Supplementary Table S1.pdf]

Supplementary Table S1. Characteristics of all included studies.

Study ID	First author, year	Country/region	Study design	Population/condition	Sample size	Rehabilitation setting	Rehabilitation exercise task	Sensor technology	Feedback strategy	Real-time feature	Main outcomes or evaluation focus	Evidence category
S1	Gamecho, 2015	ES; PT; GB	Feasibility or pilot study; System development/evaluation	Healthy elderly volunteers for rehabilitation-system validation; intended for elderly home-rehabilitation users (post-stroke/postoperative exercises referenced)	11 recruited; 10 completed	Home-based rehabilitation; Laboratory/user evaluation	Upper-limb rehabilitation; Biceps contraction/folding arm; wrist tilt left/right; 14-movement therapeutic routine	EMG; Accelerometer; Smartphone sensor; mobile robot for feedback	Biofeedback; Patient-facing feedback; Real-time feedback; animatronic robot cues; visual screen feedback comparator	Yes	Exercise monitoring; Feedback generation/support; error/interruption detection; routine completion and error counting	**Core**
S2	Mennella, 2023	IT	Algorithm/model development; Dataset study; System development/evaluation	Healthy subject/rehabilitation expert video dataset for rehabilitation-exercise validation; intended for home-based rehabilitation, older adults with frailty/sarcopenia or reduced motor ability	Dataset from one rehabilitation expert performing correct and incorrect variants of 6 exercises; no patients	Home-based rehabilitation; Telerehabilitation; Unsupervised rehabilitation; Laboratory validation	Therapeutic exercise set; resistance exercises: biceps curl, triceps curl, frontal raise, squat, leg extension, leg curl	RGB camera/computer vision; Markerless pose estimation	No feedback; assessment only; potential real-time corrective feedback to patient/therapist	Yes	Movement quality assessment; ROM-class classification; compensatory pattern recognition; valid/invalid repetition counting	**Secondary**
S3	Pereira, 2024	PT	Validation study; System development/evaluation; Cross-sectional clinical validation	Healthy volunteers/employees for shoulder rehabilitation app validation; intended for shoulder rehabilitation patients	15 participants	Home-based rehabilitation; Remote monitoring; Laboratory validation	Shoulder ROM exercise; seated shoulder external rotation with trunk rotation; standing diagonal shoulder external rotation/elevation exercise	Smartphone sensor; RGB camera/computer vision; Markerless pose estimation; Motion capture/QTM for validation	App-based feedback; Patient-facing feedback; Therapist-facing feedback; Real-time feedback	Partly; validation compared recorded outputs	Exercise quality assessment; similarity scoring; feedback generation; remote/home monitoring support	**Core**
S4	Caviedes, 2022	US	Validation study; Algorithm/model development; System development/evaluation	Healthy subjects for spine/therapeutic-exercise validation; none had back pain or were undergoing lower-back treatment	6 subjects	Laboratory validation; potential Home-based rehabilitation	Spine or trunk exercise; thoracolumbar flexion/extension, bending, rotation; single-, dual- and tri-axial motion sequences	Wearable motion sensor; Stretch/strain sensor; Camera/video for validation; proposed VR/smart-glasses visual display	Visual feedback; Biofeedback; proposed mobile/VR visual biofeedback with 3D spine model	Partly; clinical real-time biofeedback not yet tested	Joint angle/ROM estimation; 3D spine model visualization; feedback generation/support	**Core**
S5	Pan, 2017	US	Feasibility or pilot study; System development/evaluation; Within-subject repeated-measures experiment	Healthy young adults with simulated sensory deficits; intended for people with balance disorders, elderly adults and stroke survivors	15 healthy young adults	Laboratory validation; potential home/community balance rehabilitation	Balance training; Postural control training; quiet standing under no deficit, visual deficit and visual+vestibular deficit conditions	IMU; Pressure/force sensor; Vibrotactile/haptic device; portable skin-stretch sensory augmentation device	Vibrotactile/haptic feedback; Biofeedback; Real-time feedback; skin-stretch cutaneous feedback	Yes	Feedback generation/support; sensory augmentation; postural sway/balance control assessment	**Core**
S6	Zheng, 2023	CN	Algorithm/model development; Dataset study; System development/evaluation	Healthy participants and public rehabilitation-movement datasets; intended for patients performing rehabilitation exercises at home	Self-generated push-up dataset: 16 healthy males, 529 samples; IRDS: 15 patients + 14 healthy individuals; UI-PRMD: 10 healthy subjects	Home-based rehabilitation concept; Laboratory/dataset algorithm validation	Push-up assessment; public rehabilitation movements including elbow flexion, shoulder abduction, deep squat, sit-to-stand, standing shoulder scaption and related exercises	RGB camera/computer vision; Depth camera/Kinect; Motion capture/Vicon data; Skeleton/pose landmarks	Visualized feedback; color-coded skeleton joints; Patient-facing feedback potential; not a tested real-time feedback intervention	Not reported	Correct/incorrect movement classification; visualized error localization; robust skeleton-based exercise assessment	**Core**
S7	Ma, 2015	HK; CN	System development/evaluation; Within-subject experimental study	Healthy young and older adults with experimentally reduced plantar tactile sensation; intended for people with sensory deficits or fall/balance risk	30 healthy subjects: 15 older adults and 15 young adults	Laboratory validation; intended wearable/home balance training	Balance training; Static balance/Romberg test with eyes closed under no-sock, sock-off, and sock-on biofeedback conditions	Pressure/force sensor; Vibrotactile/haptic device; Wireless/Bluetooth wearable system	Vibrotactile/haptic feedback; Biofeedback; Real-time feedback	Yes	Real-time balance biofeedback; postural sway detection; vibrotactile cueing for correction	**Core**
S8	Ma, 2023	CN	System development/evaluation; Algorithm/model development; Validation study	Healthy volunteers simulating partial weight-bearing walking; intended for postoperative tibial-fracture patients during gait rehabilitation	15 healthy volunteers	Laboratory validation; intended post-discharge/home or community gait rehabilitation	Lower-limb rehabilitation; Partial weight-bearing gait training with crutches at 25%, 50%, 75% body weight and slow full weight-bearing walking	IMU; 3-axis force sensors; Instrumented shoe/insole; Audio feedback device	Auditory feedback; Biofeedback; Real-time load feedback	Yes	Tibial force estimation; real-time load monitoring; audio biofeedback for partial weight-bearing gait	**Core**
S9	Mitternacht, 2022	DE	Validation study; Device/model validation	Healthy volunteers for validation; intended for physiotherapy after knee and ankle/lower-limb injuries	5 healthy volunteers	Laboratory validation; physiotherapy-practice use case	Lower-limb rehabilitation; 10 tests: active/passive knee ROM, ankle dorsiflexion, one-leg stance, one-leg squat, drop jump, vertical jump, side hop and speedy jump	IMU; Marker-based 3D video motion analysis as reference	No explicit feedback; objective measurement/reporting for therapist and patient	Not reported	Lower-limb motion-characteristic acquisition; objective therapy-progress measurement; ROM, stability, strength and speed scoring	**Secondary**
S10	Jatesiktat, 2022	SG; TH	Algorithm/model development; Validation study; Telerehabilitation monitoring study	Healthy subjects for normative model and simulated anomalies; post-stroke subjects for qualitative evaluation; intended for daily home-based stroke rehabilitation	12 healthy subjects for normal/simulated anomaly data; 14 stroke survivors for qualitative evaluation	Home-based rehabilitation; Telerehabilitation; Laboratory validation with home-deployable sensors	Upper-limb repetitive rehabilitation exercises: rectangular drawing, tap-in-loop, hair combing and water pouring; seated with table and Kinect in front	Depth camera/Kinect; IMU; Marker-based motion capture	Therapist feedback support; No direct patient feedback; anomaly score time-series visualization	Not real-time feedback; offline/automated analysis of exercise records for therapist review	Time-series anomaly detection; segmentation/time normalization; therapist attention support for feedback generation; telerehabilitation monitoring	**Secondary**
S11	Ma, 2018	HK; CN; AU	Immediate-effect experimental study; Within-subject comparison with randomized condition order; System development/evaluation	Hemiplegic stroke patients with flexible rearfoot varus deformity at the affected side; ambulatory community-dwelling patients referred for dynamic balance disorder training	8 patients	University locomotion laboratory; overground walking on 7-m walkway; wearable system intended for future home/daily walking support	Gait training; overground walking; increase medial forefoot loading and reduce excessive foot inversion/varus during gait	Force/pressure sensor; Vibrotactile actuator; 3D motion capture; In-shoe plantar pressure system	Vibrotactile biofeedback; Real-time feedback; Patient-facing feedback; device-off condition as comparator	Yes	Real-time gait/plantar-loading monitoring; biofeedback generation; gait and plantar-pressure outcome assessment	**Core**
S12	Allum, 2021	CH	Prospective interventional repeated-measures study; biofeedback training and carry-over follow-up	Multiple sclerosis patients with subjectively reported and objectively quantified balance impairment; 13 relapsing-remitting MS and 3 primary-progressive MS	16 MS patients	University hospital/outpatient balance laboratory; supervised stance and gait balance training	Balance training; stance and gait tasks: two-leg and one-leg stance, foam stance, tandem stance/steps, walking eyes closed, walking with head pitching, barriers, 8-m walk	Gyroscope; Vibrotactile actuator; Bluetooth data transfer	Vibrotactile biofeedback; Directional head-band feedback; Real-time position- or velocity-based feedback; no-feedback follow-up to assess carry-over	Yes	Balance monitoring; Vibrotactile sensory augmentation; feedback training; carry-over effect assessment	**Core**
S13	Byl, 2015	US	Small randomized clinical trial; stratified by diagnosis; control vs kinematic-biofeedback-enhanced gait training	Adults >1 year post stroke or Parkinson's disease with gait impairments; independent in self-care and able to walk without personal assistance	24 consented/randomized; 23 completed	UCSF PT Health and Wellness Center; supervised one-on-one gait training; system designed with potential home/remote use	Progressive task-oriented gait training: walking overground/treadmill/indoor/outdoor, balance activities, stance/swing-phase practice, stairs, strengthening, flexibility, coordination and dual-task activities	Force/pressure sensor; IMU; Smart shoes; Wireless joint angle sensors; Bluetooth/ZigBee; tablet/laptop interface	Visual kinematic biofeedback; wireless real-time feedback on iPad/computer; therapist verbal feedback in both groups	Yes	Gait training feedback; visual kinematic biofeedback; functional outcome evaluation	**Core**
S14	Heo, 2026	KR	Prospective single-arm quasi-experimental pre-post study; clinical validation of AI exercise prescription app	Young adults with limited access to exercise facilities; apparently healthy; no regular resistance training in preceding 6 months; not a clinical rehabilitation patient sample	216 enrolled; 146 completed and analyzed	Smartphone-based/facility-independent training; research-facility baseline/post assessments; participants used self-selected equipment	Resistance training; 3 weekly 30-min sessions	RGB camera/computer vision; Markerless pose estimation; Smartphone app; On-device AI	Visual feedback; Textual cues; Audio feedback; color-coded skeletal overlay; gamification and progress dashboard	Yes	Exercise prescription; Real-time pose estimation; form correction; automated progression; adherence/progress monitoring	**Secondary**
S15	Argent, 2018	IE	Qualitative study; semi-structured clinician interviews with prototype demonstration; user-centred design exploration	Orthopaedic rehabilitation clinicians involved in joint replacement/TKR rehabilitation pathway; no patient participants	10 clinicians	Orthopaedic department of an acute private hospital; prototype intended for home use after TKR	Home-based post-TKR exercise support; prototype example included sitting knee extension and exercise technique/adherence monitoring; broader strengthening, ROM and function exercises	IMU; Android tablet application; Bluetooth; Avatar/visual biofeedback	Visual biofeedback; Avatar; on-screen technique feedback/advice; graphical progress tracking; potential remote clinician monitoring	Yes; study itself did not test real-time patient outcomes	Clinician acceptability/perception assessment; user-centred design input; prototype feedback-system evaluation	**Secondary**
S16	Kantan, 2023	DK; GB	User-centred system development/evaluation; qualitative focus-group evaluation; single-session feasibility study	Hemiparetic patients after stroke/brain injury; physiotherapists specializing in neurorehabilitation	Design data: 15 hemiparetic patients; focus group: 5 physiotherapists; feasibility study: 9 hemiparetic patients + 7 physiotherapists	Hospital neurorehabilitation corridor / conventional overground gait training	Lower-limb rehabilitation / hemiparetic gait training; overground walking with usual aids; swing-phase thigh and shank kinematics	Wireless IMU / inertial sensor; gyroscope; M5Stack Gray with MPU-9250 9-axis IMU; audio playback via headphones/loudspeaker	Ecological auditory feedback; splash positive reinforcement; optional negative reinforcement; delivered via wireless headphones/loudspeaker	Yes	Concurrent auditory biofeedback generation; gait-quality awareness; feasibility/usability evaluation; immediate kinematic change characterization	**Core**
S17	Chae, 2020	KR	Prospective comparative study; system development/evaluation; machine-learning exercise-recognition validation	Chronic stroke survivors with upper-limb impairment	Initially enrolled: control 10, HBR 22; completed/analyzed at 12 weeks: control 6, HBR 17 (total n=23)	Home-based rehabilitation with smartwatch/smartphone; outcome assessments at local health-care centers	Upper-limb home rehabilitation: bilateral shoulder flexion, wall push exercise, active scapular exercise, towel slide exercise	Commercial smartwatch IMU / wearable sensor; accelerometer; gyroscope; smartphone app; web/server system	App/web-based feedback and monitoring; patient could view home-exercise results; therapist remote monitoring and weekly encouragement; no explicit real-time corrective kinematic cue reported	Yes; therapist feedback was remote/asynchronous rather than continuous corrective feedback	Exercise recognition/classification; home exercise monitoring; telerehabilitation supervision; clinical/functional outcome evaluation	**Core**
S18	Sengchuai, 2022	TH	System development/evaluation; technical validation; small clinical feasibility/monitoring study	Healthy adults and rehabilitation patients: knee osteoarthritis, herniated disc, knee ligament injury, ischemic stroke, hemorrhagic stroke and Parkinson disease	6 healthy subjects + 6 patients	Laboratory testing plus hospital rehabilitation unit (Trang Hospital, Thailand)	Knee extension exercise from 0° sitting position to up to 90° extension, with ankle sandbag load; 5 repetitions/rounds with 3–6 s hold at peak position	Surface EMG; accelerometer angle sensor (GY-521); NI-myRIO embedded device; LabVIEW GUI	Visual GUI / biofeedback display of real-time EMG and ROM; therapist-facing monitoring; patient progress display	Yes	Real-time knee-extension monitoring; biofeedback/progress display; ROM and EMG measurement; therapist decision support; data logging	**Core**
S19	Martins, 2020	PT	Controlled intervention study / conference abstract; real-time biofeedback exercise-program evaluation	Older adults with moderate to high risk of falling	60 participants: experimental group n=26; control group n=34	Physiotherapy/older-adult exercise setting; control continued regular activities	Otago Exercise Program with strength and balance exercises; fall-risk reduction training	Wearable inertial sensors; pressure sensors; real-time biofeedback system	Real-time biofeedback; modality not specified in abstract	Yes	Biofeedback-supported exercise training; fall-risk outcome assessment; strength/balance training support	**Core**
S20	Jarungvittayakon, 2025	TH	Prospective randomized controlled trial; intervention study; sensor-based exergaming system evaluation	Primary mild-to-moderate knee osteoarthritis (ACR criteria; Kellgren-Lawrence stage 2–3); age 45–75 years	56 randomized; intervention n=28, control n=28; all completed 6-week follow-up	Home-based quadriceps exercise with outpatient-clinic assessment	Quadriceps strengthening exercise: deep knee flexion and full knee extension using mobile flight exergame; 5 repetitions twice daily; hold full extension 10 s	Wearable wireless IMU knee sensor; smartphone/mobile app; Wi-Fi transmitter; online cloud database	Sensor-based visual feedback through mobile exergaming; smartphone game display; app alerts/researcher reminders for missed exercise	Yes	Visual feedback/exergame control; home exercise monitoring; adherence/reminder support; clinical outcome evaluation	**Core**
S21	Sharan, 2016	IN	Randomized controlled trial; postoperative rehabilitation	Children/adolescents with cerebral palsy (spastic diplegia or quadriplegia) after	40 participants with CP; experimental	Inpatient/intensive rehabilitation setting; activity monitoring across	Gait/ambulation rehabilitation; progressive strengthening, balance and gait training,	Activity monitor / wearable accelerometer; mobile health wearable	Biofeedback; numeric activity feedback from AM; weekly therapist-	Partly	Activity monitoring; biofeedback-supported walking-target training; gait rehabilitation	**Core**

Study ID	First author, year	Country/region	Study design	Population/condition	Sample size	Rehabilitation setting	Rehabilitation exercise task	Sensor technology	Feedback strategy	Real-time feature	Main outcomes or evaluation focus	Evidence category
			intervention study	Single-Event Multilevel Lever Arm Restoration and Anti-Spasticity Surgery (SEMLARASS); GMFCS I-III	n=20, control n=20	daily ambulatory rehabilitation	cycle/treadmill training, mechanical hippotherapy and aquatic training		guided goal feedback		progress evaluation	
S22	Chen, 2022	TW	Preliminary prospective controlled trial; device reliability/validation study; telerehabilitation feasibility study	Patients after total knee arthroplasty for advanced knee osteoarthritis; additional healthy participants for reliability testing	Reliability test: 12 healthy participants; feasibility trial: 12 TKA patients (MAR n=6, HE n=6)	Home-based telerehabilitation	Post-TKA knee rehabilitation: straight leg raise, knee flexion/stretch, sit-to-stand, semi-squat on wall, standing knee flexion, hip extension/abduction, forward step-ups; 5-times sit-to-stand test	IMU; accelerometer and gyroscope; wearable motion sensor device; mobile app	Visual feedback; Mobile-app avatar feedback; Patient-facing feedback; remote clinician monitoring	Yes	Exercise monitoring; Feedback generation; Telerehabilitation; Adherence tracking; Functional performance assessment	**Core**
S23	Pang, 2025	AU	Feasibility/usability study; system development/evaluation in healthy participants	Healthy adults; system intended for future gait rehabilitation in stroke, Parkinson's disease and older adults with mobility impairments	20 healthy participants	Laboratory treadmill walking / controlled biomechanics laboratory	Treadmill gait at 2.0 km/h and 3.9 km/h under no-audio, discrete bass-tone and continuous whoosh-tone feedback conditions	IMU; 9-axis accelerometer, gyroscope and magnetometer; Bluetooth wearable; auditory feedback system	Auditory feedback; Sonification; Discrete bass tone and continuous whoosh tone delivered through speakers	Yes	Real-time gait monitoring; personalized auditory biofeedback/sonification; usability and perceptual-response evaluation	**Secondary**
S24	Lim, 2024	CA	System development/evaluation; sensor feasibility/validation study	Able-bodied adults; target application is standing-balance rehabilitation in individuals with motor incomplete spinal cord injury/disease, and potentially stroke	10 able-bodied participants	Laboratory standing-balance training setup	Visual feedback balance training with FES: hunting, color matching, ellipse and bullseye standing balance tasks; 3 sets of four 100-s exercises	Depth camera / Kinect v2; pressure mat; force plate; FES stimulator/electrodes	Visual feedback; FES assistance; cursor-target balance game feedback; alternative sensors evaluated mostly offline/passively	Partly	Sensor substitution/validation for visual feedback balance training with FES; closed-loop rehabilitation system feasibility	**Secondary**
S25	Sadarangani, 2017	CA	Controlled validation study; algorithm/model development; preliminary feasibility study	Chronic stroke survivors with mild-to-moderate upper-extremity impairment; healthy comparison group	16 participants: 8 individuals with stroke and 8 healthy participants	Controlled laboratory environment	Upper-limb reach-to-grasp tasks: 20 repetitions of reaching, grasping and moving a cup in three movement planes/directions (lateral, upward, forward)	Force myography / pressure sensing; force-sensitive resistors; validation pressure sensor	No feedback; monitoring/assessment only	No	Grasp/no-grasp classification; wearable upper-limb functional monitoring	**Core**
S26	Qi, 2025	JP	System development/evaluation; Validation study; Algorithm/model development; Cross-sectional experimental study	Community-dwelling older adults; frailty-focused chair-based exercise users; intended for older adults exercising in home/community rehabilitation settings	20 older adults	Laboratory/community-dwelling older-adult exercise assessment; intended Home-based rehabilitation	Chair-based exercise; 8 CBEs: toe/heel lifts, seated knee extension, seated leg abduction, seated knee lift, standing heel raise, standing knee bend/heel raise, standing hip abduction, high knee lifts	RGB camera/computer vision; Markerless pose estimation; sEMG	No feedback; assessment only; potential real-time visual/app feedback for home/community exercise	Partly	Movement quality assessment; correct/incorrect exercise classification; low-cost home/community exercise monitoring	**Core**
S27	Rawashdeh, 2022	US	System development/evaluation; Technical feasibility study; Preliminary clinical-trial report	Musculoskeletal rehabilitation users; preliminary trial involved outpatient physical therapy patients; proof-of-concept demonstrations used a healthy co-author	Proof-of-concept: 1 healthy individual; preliminary clinical trial: 21 physical therapy patients	Clinic-to-home rehabilitation; outpatient physical therapy clinic; home/beyond-clinic concept	Highly individualized physical therapy exercises; examples include shoulder/arm abduction, diagonal PNF-like sword-draw motion, and full knee extension; arbitrary limb-motion exercises with repetitions and hold times	IMU; Wearable motion sensor; RGB-D camera for reference validation	App-based visual feedback; Real-time feedback; Patient-facing corrective feedback; reminders/countdown/ROM path and POME error bar	Yes	Real-time corrective feedback; individualized exercise capture; repetition counting; adherence/quantity and quality monitoring	**Core**
S28	Naseer, 2025	PK; IN; IQ; JO; KR	Algorithm/model development; Dataset study; Validation study	Public/benchmark wearable-sensor physiotherapy exercise dataset; participants from source dataset performed arm and leg physical therapy exercises; actual clinical patient status NR	276,625 sensor records; source dataset involved 5 individuals performing 8 exercise classes	Offline dataset/model validation; intended home-based or remote physiotherapy support	Eight physical therapy exercise classes: extended leg raises, forward bending, straight lying-leg raises, side-lying hip abduction, alternating leg lifts prone, elbow flexion, shoulder abduction, and prone lying elbow extension	Wearable inertial/magnetic sensor; IMU	No feedback; assessment/model only	No; real-time use proposed but not evaluated	Exercise identification/classification; transfer-feature engineering for physiotherapy exercise monitoring	**Secondary**
S29	AbouGrad, 2025	GB	Algorithm/model development; Dataset study; System development/evaluation	Total knee replacement rehabilitation video dataset; primary and public physiotherapy videos with correct and intentionally flawed TKR exercises; participant demographics NR	More than 10,000 annotated video clips; over 17 h of video recordings; participant N NR	Clinical-environment videos and public repositories; intended telerehabilitation / remote patient monitoring	TKR physiotherapy exercises including leg lifts, squats and step-ups with correct and incorrect execution forms	RGB camera/video; Computer vision; Markerless pose estimation	No direct patient feedback tested; proposed app/AI feedback for remote physiotherapy monitoring	Partly	Correct/incorrect TKR exercise classification; remote patient monitoring; proposed real-time assessment and feedback	**Secondary**
S30	Kim, 2025	KR	Randomized controlled trial; Single-blind trial; Intervention study; System development/evaluation	Physically active adults with chronic ankle instability (CAI)	30 CAI participants: feedback/intervention group n=15, no-feedback control group n=15	Laboratory/supervised treadmill gait-training setting; sports medicine rehabilitation context	Walking and running gait training targeting ankle angles; treadmill-based dynamic gait modification for CAI	IMU; Wearable motion sensor; Motion capture/force plate for assessment	Visual feedback; Auditory feedback; Biofeedback; Concurrent real-time feedback	Yes	Real-time biofeedback gait retraining; gait mechanics modification; rehabilitation outcome evaluation	**Core**
S31	Schwenk, 2015	US	Randomized controlled trial; Single-blind pilot trial; Feasibility or pilot study; Intervention study	Older cancer patients with objectively confirmed chemotherapy-induced peripheral neuropathy (CIPN; VPT >25 V); balance/fall-risk rehabilitation population	22 randomized (IG n=11, CG n=11); 19 analyzed after 3 dropouts (IG n=9, CG n=10 in outcome table)	Clinic-based supervised technology-assisted balance training at University of Arizona Cancer Center; intended to support rehabilitation of CIPN-related postural control deficits	Balance training; Weight shifting; ankle point-to-point reaching in AP/ML/diagonal directions; visuomotor-rotation ankle-reaching; virtual obstacle crossing with lower-limb control	Wearable inertial sensor; IMU; Accelerometer; Gyroscope; Magnetometer; BalanSens/LegSys systems	Visual feedback; Auditory feedback; Biofeedback; Real-time lower-limb trajectory feedback; audiovisual reward/error notification	Yes	Exercise monitoring; Feedback generation/support; Balance/postural-control assessment; motor-error visualization during balance exercises	**Core**
S32	Kos, 2025	SI	Technical system development/evaluation; Bench validation study; Wearable-device engineering study	No patient or rehabilitation participants; wearable-device platform intended for real-time biomechanical feedback in sports and physical rehabilitation	4 wearable prototype devices tested; no human participants	Engineering laboratory/bench testing of wearable RTBF device and wireless streaming performance	No specific rehabilitation exercise intervention; supports motion analysis, rehabilitation monitoring and training assessment through high-frequency wearable IMU data streaming	Wearable IMU; 9-DoF sensor module; accelerometer, gyroscope, magnetometer; wireless microcontroller platform	No feedback delivered in a human study; supports real-time biofeedback	Yes	Real-time sensor-data acquisition/streaming; timing scheduler evaluation; maximum sampling frequency estimation for wearable biofeedback systems	**Secondary**
S33	Raza, 2023	PK; SA	Algorithm/model development; Dataset study; Validation study	Public multi-class skeleton-landmark dataset for physiotherapy/fitness exercise pose classification; actual patient population not reported	2,701 dataset rows/records; 133 columns; 7 exercise/pose classes	Offline machine-learning model validation; intended application in remote monitoring and physiotherapy exercise correction	Upper-limb exercise/pose classes: rest, left bicep, right bicep, left shoulder, right shoulder, left tricep, right tricep	Markerless pose/skeleton landmarks; Computer vision-derived human pose data; no wearable sensor used in this article	No direct feedback; potential AI/therapist feedback support only	No; real-time application discussed but not prospectively evaluated	Human pose estimation; physiotherapy exercise class prediction; feature selection for exercise-correction models	**Secondary**
S34	Jabri, 2022	US	Preliminary crossover study; Home-based intervention study; Feasibility or pilot study	Individuals with hereditary cerebellar ataxia (including SCA1/SCA2, ARCA1, Friedreich's ataxia, Niemann-Pick C) with balance and coordination impairments	10 recruited; 7 completed both 6-week blocks; analyses excluded missing outcomes as appropriate	Home-based balance and coordination training with laboratory assessments at A1/A2/A3	Balance training; Coordination training; static standing; standing on compliant surface; arm raises; weight shifting; gait tasks (fast/slow walking, head turns, side-stepping, high march, etc.)	Smartphone IMU; Accelerometer; Gyroscope; Vibrotactile/haptic tactors; Xsens IMU for laboratory sway assessment	Vibrotactile/haptic feedback; Smartphone app guidance; written/graphic/video guidance; feedback provided during selected repetitions	Yes	Home exercise guidance and monitoring; vibrotactile feedback generation; clinical outcome assessment; balance/coordination training support	**Core**
S35	Arens, 2021	US	Algorithm/model development; Validation study; Wearable monitoring study; Community case study	People in the chronic phase of poststroke recovery with hemiparetic gait; exosuit-assisted and unassisted walking	8 poststroke participants for in-lab validation; 1 participant returned for outdoor/community case study; 227 paretic and 220 nonparetic usable strides in validation	Overground gait laboratory with optical motion capture; outdoor/community walking case study in a busy plaza	Poststroke walking/gait training; 4-min overground bouts with exosuit active and transparent; outdoor 5-lap 150 m circuit case study; metrics related to circumduction, foot clearance and stride length	Bilateral foot IMUs; Exosuit-integrated wearable sensors; Optical motion capture and force plates for validation	No direct patient feedback tested; outputs intended for real-time movement monitoring, exosuit control and future biofeedback	Yes	Real-time gait metric estimation; movement monitoring; potential biofeedback and exosuit assistance tuning/control	**Core**
S36	Owlia, 2020	CA	Randomized controlled laboratory intervention study; Two-day training study	Healthy novice caregivers/adults with no formal caregiving or patient-handling training; no recent back pain or spine-related musculoskeletal issues	20 randomized: intervention n=10, control n=10	Simulated home environment (HomeLab, Toronto Rehabilitation Institute)	Back/lumbar movement retraining during simulated patient-handling tasks: wheelchair movement, bed-wheelchair/couch/toilet transfers, toileting-related assistance and bed preparation	IMU; Wearable motion sensor; Auditory biofeedback device (PostureCoach)	Auditory biofeedback; Training video; intermittent and continuous tones from PostureCoach	Yes	Real-time feedback generation; movement retraining; posture-risk reduction; technique correction during care tasks	**Core**
S37	Bevilacqua, 2019	IE	Algorithm/model development; Dataset validation study; Conference paper	Upper-limb rehabilitation exercises performed by healthy participants; lower-limb knee rehabilitation dataset from mixed clinical and healthy subjects	Upper-limb shoulder dataset: 29 healthy subjects; lower-limb knee dataset: 44 clinical subjects + 10 healthy subjects	Controlled data collection environment; intended application in home-based rehabilitation feedback systems	Upper-limb shoulder exercises: standing/supine shoulder flexion, abduction, internal/external rotation and active-assisted stick exercises; lower-limb knee exercises: heel slide, seated knee extension, inner range quadriceps and straight leg raise	IMU; Accelerometer; Gyroscope; Shimmer3 inertial units	No direct patient feedback tested; segmentation module intended to support autonomous biofeedback systems	Semi-online	Repetition segmentation/counting; motion-primitive detection; enabling module for autonomous exercise feedback and adherence monitoring	**Secondary**
S38	Schwenk, 2016	US	Open-label pilot randomized controlled trial	Community-dwelling outpatients with clinically confirmed amnestic mild cognitive impairment (MCI)	22 randomized: intervention n=12, control n=10; analyzed n=11 and n=9 after one unrelated medical dropout per group	Memory and Movement Disorders Center / outpatient clinic training room; future-oriented home/clinical balance training	Balance training; ankle point-to-point reaching with AP/ML/diagonal weight shifting; 20° visuomotor rotation; virtual obstacle crossing	Wearable inertial sensors; LegSys/BalanSens (BioSensics) with accelerometer, gyroscope and magnetometer; computer-based virtual interface	Visual feedback; Auditory feedback; cursor/avatar feedback; reward/error sounds	Yes	Real-time motion feedback; balance training support; movement-error visualization; clinical/functional outcome evaluation	**Core**
S39	Marusic, 2025	FR	Algorithm/model development; Dataset validation study; Conference/preprint paper	Low back pain rehabilitation exercise dataset; KERAAL includes low back pain patients aged 18–70 years, healthy subjects and trained healthy subjects simulating errors	KERAAL dataset; exact total sample not reported in uploaded manuscript; includes 12 rehabilitation patients plus healthy/simulated-error groups	Clinical/laboratory dataset recorded with Kinect V2 and Vicon; intended for in-home rehabilitation monitoring/feedback	Low back pain rehabilitation exercises: torso rotation, flank stretch and hiding face; each exercise labeled as correct or one of three common errors	Pose estimation; Depth camera/Kinect; markerless skeleton tracking; Vicon recordings available in dataset but model uses Kinect skeleton data	No direct feedback delivered; joint-importance visualization proposed to enable more detailed patient feedback	No; offline model evaluation	Error classification; movement-quality assessment; interpretable feedback support through joint-importance estimation	**Secondary**
S40	Cunha, 2025	PT	Computer-vision proof-of-concept; Pilot validation study; System development/evaluation	Rehabilitation exercise video dataset from 15 participants/patients; clinical condition not specified in uploaded article	15 participants/patients; 90 exercise videos (2	Smartphone video-based assessment with comparison to Qualisys Motion Capture System; intended for home-	Two rehabilitation exercises: Diagonal and Rotation	Smartphone RGB camera; markerless pose estimation; MediaPipe Pose Landmarker/BlazePose; Qualisys Motion	No direct feedback tested; smartphone-based independent feedback system proposed	No; authors discuss future real-time	Movement-quality assessment; accessible remote/home exercise feedback support; comparison with gold-standard motion	**Secondary**

Study ID	First author, year	Country/region	Study design	Population/condition	Sample size	Rehabilitation setting	Rehabilitation exercise task	Sensor technology	Feedback strategy	Real-time feature	Main outcomes or evaluation focus	Evidence category
					exercises × 3 repetitions × 15) plus 2 reference videos	based rehabilitation use		Capture System reference		execution as a goal	capture	
S41	Zhang, 2023	CN; GB	Algorithm/model development; System development/evaluation; Clinical measurement validation	Upper-limb injury rehabilitation; patients with left elbow or left shoulder injuries; also benchmarked on public/self-built pose-estimation datasets	Clinical study: 20 patients, randomized into Group A n=10 and Group B n=10; additional pose model training/validation used MPII, COCO, Human3.6M and self-built rehabilitation images	Clinical/laboratory rehabilitation assessment; non-contact real-time evaluation; Affiliated Hospital 2 of Nantong University ethics approval	Upper-limb rehabilitation movements involving left elbow and left shoulder; examples include elbow flexion/shoulder rehabilitation indicators and whole rehabilitation action assessment	Depth camera / RGB-D camera; Computer vision; Markerless pose estimation; physical sensors used as comparator	Assessment output only; no explicit patient-facing feedback intervention tested	Yes	Real-time pose detection; rehabilitation movement assessment; action completion and standard-reaching evaluation; comparison with standard video sequence	**Secondary**
S42	Whelan, 2017	IE	Cross-sectional analytical study; Algorithm/model development; Sensor-based technique classification	Healthy volunteers performing barbell squat; intended for lower-limb rehabilitation, resistance training and musculoskeletal injury-risk screening contexts	55 healthy volunteers; 1490 squat repetitions labelled (884 acceptable, 606 aberrant)	Laboratory; rehabilitation and injury-risk screening exercise assessment context	Barbell squat 3-repetition maximum test; acceptable technique vs natural deviations including knee valgus/varus, knees too forward, heels elevated, bent over and other deviations	IMU; Shimmer wearable sensors; accelerometer, gyroscope, magnetometer	No feedback; assessment only; potential future exercise biofeedback application	No; offline model validation, with potential for future feedback applications	Binary classification of acceptable vs aberrant squat technique; multi-class classification of exact deviations; comparison of personalised vs global classifiers	**Secondary**
S43	Whelan, 2017	IE	Cross-sectional analytical study; Algorithm/model development; Sensor-based technique classification	Healthy volunteers performing single-leg squat; intended for lower-limb rehabilitation and injury-risk screening	83 healthy volunteers; 10 single-leg squat repetitions each (approximately 830 repetitions; dataset included 52 acceptable and 778 aberrant repetitions)	Laboratory; rehabilitation/injury-risk screening exercise assessment	Left-leg single-leg squat; overall acceptable vs aberrant movement plus deviations in trunk, pelvis, knee, foot pronation, oscillation and loss of balance	IMU; Shimmer wearable sensors; accelerometer, gyroscope, magnetometer	No feedback; assessment only; authors note potential biofeedback application	No; offline model validation	Overall SLS technique classification; specific deviation detection; potential input to exercise biofeedback	**Secondary**
S44	Kent, 2015	AU; DK	Pilot randomized controlled trial; Multicentre cluster-randomised placebo-controlled trial	Adults with sub-acute or chronic low back pain or back-related leg pain in primary care	112 patients recruited; Movement Biofeedback n=58; Guidelines-based Care/placebo n=54; 8 clinics/clusters	Primary care hospitals/outpatient clinics in Victoria, Australia; clinic sessions plus activities of daily living	Individualised lumbopelvic movement/posture retraining; live movement training; daily-life posture and movement modification; supplementary patient-specific exercises	Wearable motion sensors; surface EMG; ViMove system with audio/vibration recording device	Real-time feedback; Biofeedback; on-screen visual feedback in clinic; audio beeps and/or vibration alerts during daily activities	Yes	Movement/posture assessment; individualized biofeedback-rule programming; clinical outcome evaluation; movement retraining support	**Core**
S45	van der Logt, 2016	CH	Crossover study; within-subject biofeedback training experiment	People with multiple sclerosis with balance impairment; 6 relapsing-remitting MS, 2 primary progressive MS, 2 secondary progressive MS	10 MS patients completed crossover study; 2 additional patients dropped out after first visit due to scheduling conflicts	Clinical/laboratory balance assessment and training at University Hospital Basel; stance and gait tasks	Balance and gait tasks: stance on one/two legs, eyes open/closed, foam surface, tandem stance, tandem walking, walking over barriers, 8 m eyes-open walking and 3 m eyes-closed walking	Body-worn gyroscopes; Vibrotactile feedback device; SwayStar and BalanceFreedom	Vibrotactile biofeedback; direction-specific head-mounted feedback; angle feedback for stance and angular-velocity feedback for gait	Yes	Real-time balance biofeedback; assessment of immediate training and carry-over effects on stance/gait sway	**Core**
S46	Candreia, 2023	CH	Retrospective intervention study; pre-post balance training evaluation	Patients with persistent postural-perceptual dizziness (PPPD); subgroups with quantified balance deficit (QBD) or dizziness only (DO)	23 PPPD patients: 12 QBD and 11 DO	Clinical balance laboratory / vestibular rehabilitation setting	Balance training; 14 stance and gait assessment tasks; 11 trained tasks including standing eyes closed/on foam, tandem walking, walking with head movements, stairs/barriers	Gyroscope; wearable vibro-tactile feedback device; SwayStar™ + BalanceFreedom™	Vibro-tactile biofeedback; headband directional feedback when sway exceeded threshold	Yes	Balance assessment; real-time vibro-tactile biofeedback; rehabilitation progress evaluation	**Core**
S47	Costantini, 2018	IT; US	Technical note; system development/evaluation; within-subject validation study	Healthy young and older adults; potential users for balance/postural rehabilitation, not actual patients	10 healthy subjects: 6 older and 4 young	Laboratory standing-balance testing	Standing balance tasks under four conditions: floor/foam surface × eyes open/closed	IMU; accelerometer; wireless audio-biofeedback system; headphones	Auditory biofeedback; pleasant/no-warning sound and increasingly aversive warning sounds via headphones	Yes	Real-time balance monitoring; audio-feedback generation; balance assistance	**Secondary**
S48	Liu, 2025	GB	Device validation study; system development/evaluation	Healthy participants performing knee-extension tasks; intended for home-based musculoskeletal rehabilitation	15 healthy participants	Laboratory validation with intended home-based rehabilitation use	Knee extension exercises: static MVIC at 65°, 90°, 115°, 140°; dynamic knee flexion/extension under five resistance-band loads	sEMG; IMU/accelerometer; force gauge; optical motion capture for validation	Potential visual/audio/game-based biofeedback via mobile app/AI-enabled game; feedback not the primary tested intervention	Partly	Kinematic and muscle-activation monitoring; feasibility validation for feedback-supported knee-extension rehabilitation	**Secondary**
S49	Bell, 2019	US	Device validation and usability study; system development/evaluation	Healthy control participants; intended for remote management of knee rehabilitation after injury/surgery	10 healthy participants recruited; kinematic analysis excluded 1 participant due to procedural error	Controlled laboratory with mobile app and intended telerehabilitation/remote monitoring context	Knee rehabilitation exercises: heel slides, short arc quadriceps contractions, sit-to-stand; 10 repetitions each	IMU; two wireless IMUs; mobile application; web-based clinician portal; OptiTrack validation system	Visual feedback; mobile-app animation, numerical knee angle, color-coded red/yellow/green target zone	Yes	Remote knee rehabilitation monitoring; real-time visual feedback; adherence/performance tracking	**Secondary**
S50	Chen, 2017	NZ	Feasibility study; crossover laboratory gait-retraining experiment; system development/evaluation	Healthy adults; intended future application to knee osteoarthritis gait retraining	10 healthy subjects	Motion-analysis laboratory; treadmill gait retraining	Gait retraining during treadmill walking: modifying foot progression angle (FPA) and step width (SW)	Haptic/vibrotactile device; Vicon motion capture; force-instrumented treadmill; Arduino-controlled eccentric rotating mass motors	Haptic feedback; binary vibrotactile feedback and tactile apparent movement delivered through ankle bracelet	Yes	Real-time haptic feedback for multi-parameter gait retraining	**Secondary**
S51	Chen, 2020	TW; HK	Pilot feasibility study; Reliability/validation study; Prospective controlled pilot trial; System development/evaluation	Patients with shoulder adhesive capsulitis (frozen shoulder); healthy participants used for shoulder-ROM reliability testing	Reliability: 10 healthy participants + 15 patients with AC; intervention: 15 AC patients allocated to sensor-assisted (n=8) or home-exercise (n=7), final analysis 7 vs 7 after 1 exclusion	Home-based rehabilitation; Telerehabilitation; monthly outpatient/physician follow-up	Upper-limb/shoulder rehabilitation: shoulder ROM measurement; Codman/pendulum exercises; wall-walking stretch; cane stretches for shoulder flexion, extension, abduction, internal rotation and external rotation	IMU; wearable 6-axis IMU sensors (accelerometer + gyroscope); mobile apps for patient and physician	App-based visual feedback; 3D avatar mirroring; target-angle achievement feedback; clinician text messages and remote supervision	Yes	Shoulder ROM assessment; home-exercise guidance; real-time exercise mirroring/feedback; adherence/progress monitoring; remote clinician supervision	**Core**
S52	Kos, 2019	SI	Technical case study; System architecture and prototype evaluation; Feasibility study	Elite/healthy swimmer field-test data; intended users are rehabilitation or injury-prevention swimmers/patients under therapist supervision, not a clinical patient sample	Field case study using an elite professional swimmer; no rehabilitation-patient sample reported	Swimming pool; therapist-supervised rehabilitation/physical exercise monitoring setting	Swimming rehabilitation exercise: four-lap sequence with butterfly, backstroke, breaststroke and front crawl; additional backstroke/front-crawl laps at low, medium and high intensity	Waterproof wearable IMU; 6DoF inertial sensor device with 3D accelerometer and 3D gyroscope; real-time processing/therapist feedback application	Therapist-facing real-time feedback; therapist can provide oral or other corrective feedback to user; autonomous/cloud feedback architectures proposed	Yes	Real-time therapist feedback; swimming exercise monitoring; stroke-style detection; quantitative movement/symmetry evaluation	**Secondary**
S53	Carpinella, 2017	IT	Randomized controlled trial; Pilot clinical trial; Rehabilitation biofeedback intervention	Parkinson's disease patients with balance/gait impairment; Hoehn-Yahr stage 2-4	42 PD subjects randomized; analyzed post-treatment EG n=17 and CG n=20; 5 discontinued training and 5 lost at follow-up	Clinical rehabilitation gym	Balance and gait training: weight shifting, trunk/posture control, sit-to-stand, gait initiation, step tasks, straight-line walking, walking with turns/obstacles, dual-task/proprioceptive challenges	IMU; six wearable inertial sensors (3D accelerometer, gyroscope, magnetometer) plus PC/LCD Gamepad system; force platform for stabilometry outcomes	Visual feedback; Auditory feedback; positive/negative feedback; walking tasks used auditory feedback only; fading schedule	Yes	Biofeedback-based balance/gait rehabilitation; movement feedback; performance scoring; clinical outcome evaluation	**Core**
S54	Argent, 2019	IE	Mixed-methods user evaluation; Feasibility/usability study; System development/evaluation	Orthopaedic rehabilitation patients after knee replacement surgery (TKR or unicompartmental knee replacement)	15 knee-replacement patients: acute group n=10, post-acute group n=5	Home-based rehabilitation; private hospital and home follow-up visits	Post-operative knee rehabilitation exercises prescribed by physiotherapist; app example includes straight leg raise; lower-limb exercise sessions with avatar, repetition counting and technique feedback	IMU; single Shimmer3 wearable IMU with tri-axial accelerometer and gyroscope; Android tablet application	App-based visual biofeedback; real-time avatar mirroring; repetition counter; post-exercise technique advice; progress/adherence statistics	Yes	Exercise biofeedback; real-time avatar mirroring; repetition counting; technique feedback; adherence/progress tracking; patient-reported outcome capture	**Core**
S55	Martins, 2020	PT	Conference abstract; Technical feasibility study; System development/evaluation	Older adults participating in fall-prevention physiotherapy exercise testing	16 older adults	Physiotherapy clinic	Five exercises from the Otago Exercise Program focused on strength and balance for fall prevention	IMU; two wearable inertial sensors; pressure/force platform	Quantitative biofeedback; clinic/therapist-facing exercise feedback; personalized exercise feedback concept	Yes	Exercise tracking; quantitative biofeedback generation; individualized fall-prevention exercise support	**Core**

Notes. Study IDs correspond to the identifiers used in the Results section. “NR” indicates that the information was not reported or was insufficiently described in the available full text or abstract. Evidence category was assigned for synthesis purposes only: “Core” indicates studies directly involving rehabilitation patients, disease populations, or clearly defined rehabilitation contexts, including rehabilitation-oriented feedback systems applied in physiotherapy or fall-prevention settings; “Secondary” indicates healthy-participant validation, algorithm/model validation, technical prototype evaluation, conference abstracts, or indirect rehabilitation-support evidence when not directly linked to a rehabilitation intervention or setting.

Evidence category check: Core = 33; Secondary = 22; Total = 55.
