# Supplementary material for: Sensor-Based Movement Quality Assessment and Biofeedback for Rehabilitation Exercise: A Scoping Review with Implications for Home-Based and Remote Rehabilitation
Source: Healthcare (Basel). 2026 Aug 7;14(16):2450. doi: 10.3390/healthcare14162450 (PMC13512171; doi:10.3390/healthcare14162450)
Supplement: Supplementary file 1 [file healthcare-14-02450-s001.zip › Supplementary Table S2.pdf]

Supplementary Table S2. Search strategies used for the scoping review.

Source	Search strategy
**PubMed/MEDLINE**	**Date searched:** [31 May 2026] **Search 1: Sensor-based rehabilitation exercise and movement-quality assessment** ((rehabilitation[Title/Abstract] OR rehabilitative[Title/Abstract] OR physiotherapy[Title/Abstract] OR “physical therapy”[Title/Abstract] OR “therapeutic exercise”[Title/Abstract] OR “rehabilitation exercise”[Title/Abstract] OR “home exercise”[Title/Abstract] OR “home-based exercise”[Title/Abstract] OR “exercise therapy”[Title/Abstract]) AND (“wearable sensor”[Title/Abstract] OR “wearable sensors”[Title/Abstract] OR “motion sensor”[Title/Abstract] OR “motion sensors”[Title/Abstract] OR “inertial measurement unit”[Title/Abstract] OR IMU[Title/Abstract] OR accelerometer[Title/Abstract] OR gyroscope[Title/Abstract] OR magnetometer[Title/Abstract] OR “smartphone sensor”[Title/Abstract] OR “mobile sensor”[Title/Abstract] OR “depth camera”[Title/Abstract] OR “markerless motion capture”[Title/Abstract] OR “pose estimation”[Title/Abstract] OR “surface electromyography”[Title/Abstract] OR sEMG[Title/Abstract] OR EMG[Title/Abstract] OR “pressure sensor”[Title/Abstract] OR “force sensor”[Title/Abstract]) AND (“movement quality”[Title/Abstract] OR “movement assessment”[Title/Abstract] OR “motion assessment”[Title/Abstract] OR “exercise assessment”[Title/Abstract] OR “exercise evaluation”[Title/Abstract] OR “exercise technique”[Title/Abstract] OR “movement performance”[Title/Abstract] OR “exercise performance”[Title/Abstract] OR “range of motion”[Title/Abstract] OR “joint angle”[Title/Abstract] OR “movement velocity”[Title/Abstract] OR “postural stability”[Title/Abstract] OR symmetry[Title/Abstract] OR “compensatory movement”[Title/Abstract] OR “repetition quality”[Title/Abstract] OR “exercise recognition”[Title/Abstract] OR “activity recognition”[Title/Abstract])) Filters: English; publication date from 2014/01/01 to 2026/05/31. **Search 2: Rehabilitation exercise feedback, biofeedback, and remote rehabilitation** ((rehabilitation[Title/Abstract] OR rehabilitative[Title/Abstract] OR physiotherapy[Title/Abstract] OR “physical therapy”[Title/Abstract] OR “therapeutic exercise”[Title/Abstract] OR “rehabilitation exercise”[Title/Abstract] OR “home exercise”[Title/Abstract] OR “home-based rehabilitation”[Title/Abstract] OR telerehabilitation[Title/Abstract] OR “remote rehabilitation”[Title/Abstract]) AND (“wearable sensor”[Title/Abstract] OR “wearable sensors”[Title/Abstract] OR “motion sensor”[Title/Abstract] OR “inertial measurement unit”[Title/Abstract] OR IMU[Title/Abstract] OR accelerometer[Title/Abstract] OR gyroscope[Title/Abstract] OR smartphone[Title/Abstract] OR “mobile app”[Title/Abstract] OR “mobile application”[Title/Abstract] OR “depth camera”[Title/Abstract] OR “pose estimation”[Title/Abstract] OR “surface electromyography”[Title/Abstract] OR EMG[Title/Abstract] OR “pressure sensor”[Title/Abstract] OR “force sensor”[Title/Abstract]) AND (biofeedback[Title/Abstract] OR feedback[Title/Abstract] OR “real-time feedback”[Title/Abstract] OR “visual feedback”[Title/Abstract] OR “auditory feedback”[Title/Abstract] OR “haptic feedback”[Title/Abstract] OR “vibrotactile feedback”[Title/Abstract] OR “exercise feedback”[Title/Abstract] OR “training feedback”[Title/Abstract] OR “exercise guidance”[Title/Abstract] OR “exercise monitoring”[Title/Abstract] OR “exercise progression”[Title/Abstract] OR “remote monitoring”[Title/Abstract])) Filters: English; publication date from 2014/01/01 to 2026/05/31.
**Web of Science Core Collection**	**Date searched:** [31 May 2026] **Search 1: Sensor-based rehabilitation exercise and movement-quality assessment** TS = ((rehabilitation OR rehabilitative OR physiotherapy OR “physical therapy” OR “therapeutic exercise” OR “rehabilitation exercise” OR “home exercise” OR “home-based exercise” OR “exercise therapy”) AND (“wearable sensor*” OR ”motion sensor*” OR “inertial measurement unit” OR IMU OR accelerometer OR gyroscope OR magnetometer OR “smartphone sensor*” OR ”mobile sensor*” OR “depth camera” OR “markerless motion capture” OR “pose estimation” OR “surface electromyography” OR sEMG OR EMG OR “pressure sensor*” OR ”force sensor*”) AND (“movement quality” OR “movement assessment” OR “motion assessment” OR “exercise assessment” OR “exercise evaluation” OR “exercise technique” OR “movement performance” OR “exercise performance” OR “range of motion” OR “joint angle” OR “movement velocity” OR “postural stability” OR symmetry OR “compensatory movement” OR “repetition quality” OR “exercise recognition” OR “activity recognition”)) Refined by: Language = English; Publication years = 2014–2026. **Search 2: Rehabilitation exercise feedback, biofeedback, and remote rehabilitation** TS = ((rehabilitation OR rehabilitative OR physiotherapy OR “physical therapy” OR “therapeutic exercise” OR “rehabilitation exercise” OR “home exercise” OR “home-based rehabilitation” OR telerehabilitation OR “remote rehabilitation”) AND (“wearable sensor*” OR ”motion sensor*” OR “inertial measurement unit” OR IMU OR accelerometer OR gyroscope OR smartphone OR “mobile app” OR “mobile application” OR “depth camera” OR “pose estimation” OR “surface electromyography” OR EMG OR “pressure sensor*” OR ”force sensor*”) AND (biofeedback OR feedback OR “real-time feedback” OR “visual feedback” OR “auditory feedback” OR “haptic feedback” OR “vibrotactile feedback” OR “exercise feedback” OR “training feedback” OR “exercise guidance” OR “exercise monitoring” OR “exercise progression” OR “remote monitoring”)) Refined by: Language = English; Publication years = 2014–2026.
**IEEE Xplore**	**Date searched:** [31 May 2026] Because IEEE Xplore has practical limitations for very long Boolean strings and because engineering-oriented studies are indexed with heterogeneous terminology, multiple grouped searches were used. Searches were limited to English-language records published from 2014 to 2026. **Search 1: Inertial sensors and rehabilitation exercise assessment** (“rehabilitation” OR “physical therapy” OR “physiotherapy”) AND (“inertial measurement unit” OR IMU OR accelerometer OR gyroscope) AND (“exercise assessment” OR “movement assessment” OR “exercise monitoring” OR “movement quality”) **Search 2: Wearable sensors and rehabilitation feedback** (“rehabilitation” OR “therapeutic exercise” OR “home exercise”) AND (“wearable sensor” OR “wearable sensors” OR “motion sensor” OR “motion sensors”) AND (biofeedback OR feedback OR “real-time feedback” OR “exercise guidance”) **Search 3: Pose estimation and rehabilitation exercise monitoring** (“rehabilitation” OR “physical therapy” OR “home-based rehabilitation”) AND (“pose estimation” OR “markerless motion capture” OR “depth camera” OR “RGB-D” OR Kinect) AND (“exercise assessment” OR “exercise evaluation” OR “exercise monitoring” OR “movement quality”) **Search 4: Electromyography and rehabilitation exercise feedback** (“rehabilitation” OR “therapeutic exercise” OR “physical therapy”) AND (EMG OR sEMG OR “surface electromyography”) AND (biofeedback OR feedback OR “exercise monitoring” OR “exercise assessment”) **Search 5: Pressure or force sensors and rehabilitation training feedback** (“rehabilitation” OR “physical therapy” OR “gait training” OR “balance training”) AND (“pressure sensor” OR “force sensor” OR “force plate” OR

Source	Search strategy
	“pressure platform” OR “plantar pressure”) AND (feedback OR biofeedback OR monitoring OR assessment) **Search 6: Telerehabilitation and sensor-based exercise feedback** (telerehabilitation OR “remote rehabilitation” OR “home-based rehabilitation”) AND (“wearable sensor” OR IMU OR accelerometer OR “motion sensor” OR smartphone OR “mobile app”) AND (feedback OR biofeedback OR “exercise monitoring” OR “exercise guidance”)
**Google Scholar**	**Date searched:** [31 May 2026] Google Scholar was used as a supplementary search source. Searches were performed using highly relevant keyword combinations, and the first 50 records sorted by relevance were screened for each search string. The following keyword combinations were used: 1. “wearable sensor” “rehabilitation exercise” biofeedback 2. “IMU” “rehabilitation exercise” feedback 3. “sensor-based” “movement quality” rehabilitation 4. “home-based rehabilitation” “exercise monitoring” sensor 5. “telerehabilitation” “sensor feedback” exercise 6. “pose estimation” “rehabilitation exercise” assessment 7. “movement assessment” “therapeutic exercise” “wearable sensor” 8. “exercise technique” rehabilitation “biofeedback” sensor
**Search limits applied across sources**	Publication date: January 2014 to May 2026. Language: English. Population/context: human rehabilitation, therapeutic exercise, rehabilitation-oriented validation, home-based rehabilitation, telerehabilitation, or rehabilitation-related movement-quality assessment. Excluded at screening: studies focused only on general physical activity monitoring, sport performance, non-rehabilitation fitness tracking, diagnosis-only applications, non-human studies, reviews, protocols, editorials, opinions, patents, and studies without extractable information relevant to sensor-based rehabilitation exercise assessment, biofeedback, or guidance.

Notes. Search terms were adapted to the syntax and indexing structure of each database. PubMed/MEDLINE and Web of Science Core Collection used two complementary strategies: one focused on sensor-based movement-quality assessment and one focused on rehabilitation feedback, biofeedback, and remote rehabilitation. IEEE Xplore was searched using grouped shorter strategies because of the heterogeneity of engineering terminology and the practical limitations of long Boolean queries. Google Scholar was used only as a supplementary source, and records were screened by relevance ranking.
