# Supplementary material for: Sensor-Based Movement Quality Assessment and Biofeedback for Rehabilitation Exercise: A Scoping Review with Implications for Home-Based and Remote Rehabilitation
Source: Healthcare (Basel). 2026 Aug 7;14(16):2450. doi: 10.3390/healthcare14162450 (PMC13512171; doi:10.3390/healthcare14162450)
Supplement: Supplementary file 1 [file healthcare-14-02450-s001.zip › Supplementary Table S3.pdf]

Supplementary Table S3. Sensor technologies, placements, input signals, and reference standards used in the included studies.

Sensor or technology category	Typical devices or technologies	Common placement/setup	Input signals/data	Common reference standards or comparators	Main rehabilitation uses	No. of studies	Representative studies
**Inertial sensors**	IMU, accelerometer, gyroscope, magnetometer, wearable motion sensor, smartwatch inertial sensor, smartphone inertial sensor	Trunk, waist, lower back, thigh, shank, foot, ankle, knee region, wrist, upper arm, sternum, affected limb	Linear acceleration, angular velocity, orientation, Euler angles, quaternion data, joint angle estimates, motion trajectories, repetition cycles	Optical motion capture, goniometer, force platform, therapist observation, manually labelled repetitions, clinical performance tests	ROM estimation, gait monitoring, balance assessment, trunk sway monitoring, exercise repetition detection, movement-quality feedback, home exercise tracking	43	(Gamecho et al., 2015 , Burns et al., 2018 , Caviedes et al., 2022 , Pan et al., 2016 , Ma et al., 2023 , Mitternacht et al., 2022 , Jatesiktat et al., 2022 , Allum et al., 2021 , Byl et al., 2015 , Heo et al., 2026 , Argent et al., 2018 , Kantan et al., 2023 , Chae et al., 2020 , Sengchuai et al., 2022 , Martins et al., 2020 , Jarungvittayakon et al., 2025 , Sharan et al., 2016 , Chen et al., 2022 , Pang et al., 2025 , Lim et al., 2024 , Rawashdeh et al., 2022 , Naseer et al., 2025 , Kim et al., 2025 , Schwenk et al., 2016a , Kos et al., 2025 , Jabri et al., 2022 , Arens et al., 2021 , Owlia et al., 2020 , Bevilacqua et al., 2019 , Schwenk et al., 2016b , Whelan et al., 2017 , Whelan et al., 2016 , Kent et al., 2015 , van der Logt et al., 2016 , Candreia et al., 2023 , Costantini et al., 2018 , Liu et al., 2025 , Bell et al., 2019 , Chen et al., 2020 , Kos and Umek, 2019 , Carpinella et al., 2016 , Argent et al., 2019 , Martins and Guia, 2020)
**Vision-based and markerless motion capture systems**	RGB camera, depth camera, Kinect-like sensor, markerless motion capture, pose estimation, skeleton tracking, video-based assessment	Camera positioned in front of or around the participant; whole-body or segmental tracking; home or laboratory video setup	RGB/depth images, 2D or 3D keypoints, skeleton coordinates, joint trajectories, posture features, video frames	Expert annotation, clinician/therapist labels, optical motion capture, pre-labelled datasets, exercise correctness labels	Exercise recognition, movement-quality classification, remote rehabilitation assessment, posture estimation, home exercise monitoring, automatic error detection	18	(Mennella et al., 2023 , Burns et al., 2018 , Caviedes et al., 2022 , Zheng et al., 2023 , Mitternacht et al., 2022 , Jatesiktat et al., 2022 , Heo et al., 2026 , Lim et al., 2024 , Qi et al., 2025 , Rawashdeh et al., 2022 , AbouGrad et al., 2025 , Kim et al., 2025 , Raza et al., 2023 , Arens et al., 2021 , Marusic et al., 2025 , Cunha et al., 2025 , Zhang et al., 2023 , Kos and Umek, 2019)

**Pressure, force, and load sensors**	Pressure platform, force plate, plantar pressure sensor, force-sensitive resistor, force gauge, smart insole, load-monitoring system, force myography sensor	Under feet, shoe/insole, force platform, hand or grasp interface, ankle or limb-mounted load system, resistance-band or task-specific setup	Center of pressure, ground reaction or plantar pressure, weight distribution, loading symmetry, force output, partial weight-bearing data, grasp or muscle-force proxy signals	Force platform, clinical balance tests, therapist judgement, prescribed load targets, motion capture, dynamometer or force gauge	Balance feedback, partial weight-bearing training, gait loading feedback, fall-prevention exercise, grasp monitoring, knee-extension force assessment	15	(Pan et al., 2016 , Ma et al., 2015 , Ma et al., 2023 , Ma et al., 2018 , Byl et al., 2015 , Kantan et al., 2023 , Martins et al., 2020 , Lim et al., 2024 , Sadarangani et al., 2017 , Kim et al., 2025 , Arens et al., 2021 , Liu et al., 2025 , Chen et al., 2017 , Carpinella et al., 2016 , Martins and Guia, 2020)
**Electromyography and muscle-activity sensors**	Surface EMG, low-cost sEMG prototype, EMG biofeedback sensor, EMG envelope acquisition	Biceps, quadriceps, target muscle group, limb-specific electrode placement with reference electrode	Raw EMG, rectified EMG, EMG envelope, normalized muscle activation, MVC-normalized activity	MVC, clinical task execution, motion capture, force measurement, therapist-defined task targets	Muscle activation monitoring, exercise correctness detection, knee-extension training support, muscle contraction feedback, home-based biofeedback	5	(Gamecho et al., 2015 , Sengchuai et al., 2022 , Qi et al., 2025 , Kent et al., 2015 , Liu et al., 2025)
**Mobile, web, and cloud-based rehabilitation platforms**	Smartphone app, tablet app, web portal, clinician dashboard, cloud database, remote monitoring platform, app-based avatar or training interface	Patient-side mobile device; therapist-side portal or app; cloud-based synchronization; home or remote rehabilitation setup	Exercise completion logs, sensor-derived metrics, patient-reported outcomes, pain scores, adherence data, feedback history, progress records	Usability questionnaires, therapist review, patient self-report, clinical outcome measures, system logs	Home exercise support, telerehabilitation, adherence monitoring, remote therapist supervision, individualized task assignment, progress visualization	31	(Gamecho et al., 2015 , Mennella et al., 2023 , Burns et al., 2018 , Caviedes et al., 2022 , Pan et al., 2016 , Ma et al., 2015 , Byl et al., 2015 , Heo et al., 2026 , Argent et al., 2018 , Chae et al., 2020 , Jarungvittayakon et al., 2025 , Sharan et al., 2016 , Chen et al., 2022 , Lim et al., 2024 , Qi et al., 2025 , Rawashdeh et al., 2022 , Naseer et al., 2025 , AbouGrad et al., 2025 , Raza et al., 2023 , Jabri et al., 2022 , Bevilacqua et al., 2019 , Cunha et al., 2025 , Zhang et al., 2023 , Whelan et al., 2017 , Whelan et al., 2016 , Liu et al., 2025 , Bell et al., 2019 , Chen et al., 2017 , Chen et al., 2020 , Kos and Umek, 2019 , Argent et al., 2019)
**Multisensor or hybrid systems**	IMU + EMG, IMU + pressure/force	Multi-segment body placement; sensor	Combined kinematic, kinetic,	Optical motion capture, clinical	Multidimensional movement-quality	15	(Gamecho et al., 2015 , Pan et al., 2016 , Ma et al., 2023 , Byl et al., 2015 , Sengchuai et al., 2022 , Jarungvittayakon

	sensor, IMU + mobile app, IMU + optical motion capture, sensor + avatar/game interface, wearable sensor + therapist portal	fusion across trunk, limbs, foot, or clinical interface; combined patient and therapist systems	muscle-activity, task-performance, adherence, and patient-reported data	assessments, therapist labels, goniometry, force platform, patient-reported outcomes	assessment, biofeedback generation, rehabilitation progress monitoring, remote decision support		et al., 2025 , Chen et al., 2022 , Kim et al., 2025 , Jabri et al., 2022 , Liu et al., 2025 , Bell et al., 2019 , Chen et al., 2020 , Carpinella et al., 2016 , Argent et al., 2019 , Martins and Guia, 2020)
**Haptic, vibrotactile, robotic, or actuator-based feedback components**	Vibrotactile actuator, haptic bracelet, tactile apparent movement device, wearable vibration unit, animatronic/robotic feedback device	Trunk, waist, lower limb, ankle, foot, or external robotic interface depending on task	Sensor-derived deviation from target posture, gait parameter, balance threshold, or movement state translated into tactile/robotic cues	Target movement parameters, therapist-defined thresholds, motion capture, clinical task performance	Balance correction, gait retraining, trunk sway feedback, foot progression angle and step-width training, engagement support	10	(Gamecho et al., 2015 , Pan et al., 2016 , Ma et al., 2015 , Ma et al., 2018 , Allum et al., 2021 , Jabri et al., 2022 , Kent et al., 2015 , van der Logt et al., 2016 , Candreia et al., 2023 , Chen et al., 2017)

Notes. Categories are not mutually exclusive because many systems combined multiple sensors, mobile applications, therapist dashboards, or feedback actuators. The “No. of studies” column therefore indicates how many studies involved each technology category rather than mutually exclusive totals. “Mobile, web, and cloud-based rehabilitation platforms” were included as a technology layer because they frequently mediated data visualization, patient feedback, therapist supervision, and remote rehabilitation management. ROM, range of motion; IMU, inertial measurement unit; EMG, electromyography; sEMG, surface electromyography; MVC, maximum voluntary contraction.
