# Supplementary material for: Sensor-Based Movement Quality Assessment and Biofeedback for Rehabilitation Exercise: A Scoping Review with Implications for Home-Based and Remote Rehabilitation
Source: Healthcare (Basel). 2026 Aug 7;14(16):2450. doi: 10.3390/healthcare14162450 (PMC13512171; doi:10.3390/healthcare14162450)
Supplement: Supplementary file 1 [file healthcare-14-02450-s001.zip › Supplementary Table S4.pdf]

Supplementary Table S4. Movement-quality constructs and computational approaches used in the included studies.

Movement-quality construct	Operational metrics or extracted features	Typical rehabilitation tasks	Common sensor inputs	Computational approaches	No. of studies	Representative studies
**Exercise correctness, technique quality, and error classification**	Correct/incorrect repetition, exercise form quality, movement-quality score, error type, error localization, abnormal movement phase, movement dysfunction, pass/fail against predefined targets	Home exercise, knee rehabilitation exercise, shoulder exercise, chair-based exercise, squat, lunge, upper-limb rehabilitation, video-based rehabilitation tasks	IMU, smartphone sensor, RGB/depth camera, pose-estimation keypoints, sEMG, mobile-app logs	Threshold rules, state-machine repetition counting, clinician-labelled classification, SVM, random forest, CNN, ST-GCN, Transformer-based skeleton model, pose-estimation model, DTW/cosine similarity	27	(Gamecho et al., 2015 , Mennella et al., 2023 , Burns et al., 2018 , Caviedes et al., 2022 , Zheng et al., 2023 , Jatesiktat et al., 2022 , Heo et al., 2026 , Argent et al., 2018 , Chae et al., 2020 , Sengchuai et al., 2022 , Jarungvittayakon et al., 2025 , Chen et al., 2022 , Sadarangani et al., 2017 , Qi et al., 2025 , Rawashdeh et al., 2022 , Naseer et al., 2025 , AbouGrad et al., 2025 , Bevilacqua et al., 2019 , Marusic et al., 2025 , Cunha et al., 2025 , Zhang et al., 2023 , Whelan et al., 2017 , Whelan et al., 2016 , Bell et al., 2019 , Chen et al., 2020 , Argent et al., 2019 , Martins and Guia, 2020)
**Range of motion, joint angle, and segmental kinematics**	Joint angle, ROM, flexion/extension angle, abduction/adduction angle, trunk inclination, lumbar flexion exposure, knee valgus, angular velocity, Euler angles, quaternion-derived relative angles	Knee flexion/extension, short arc quadriceps exercise, sit-to-stand, shoulder ROM exercise, gait training, spine posture control, balance tasks	IMU, accelerometer, gyroscope, magnetometer, stretch sensor, smartphone sensor, depth/RGB camera, pose-estimation keypoints	Sensor-fusion orientation estimation, quaternion/Euler-angle calculation, anatomical alignment, vector-based joint-angle estimation, pose-estimation algorithms, threshold comparison, Bland–Altman/RMSE/ICC validation	25	(Mennella et al., 2023 , Burns et al., 2018 , Caviedes et al., 2022 , Mitternacht et al., 2022 , Allum et al., 2021 , Byl et al., 2015 , Kantan et al., 2023 , Chae et al., 2020 , Sengchuai et al., 2022 , Jarungvittayakon et al., 2025 , Chen et al., 2022 , Qi et al., 2025 , Rawashdeh et al., 2022 , Kim et al., 2025 , Arens et al., 2021 , Owlia et al., 2020 , Cunha et al., 2025 , Zhang et al., 2023 , Kent et al., 2015 , van der Logt et al., 2016 , Liu et al., 2025 , Bell et al., 2019 , Chen et al., 2020 , Carpinella et al., 2016 , Martins and Guia, 2020)
**Postural stability, balance control, and trunk sway**	Trunk sway angle, angular velocity, COP/COM displacement, sway range, sway variance, balance control index, postural stability, weight shifting, step-outs, perceived stability	Static standing, foam-surface standing, tandem stance, balance training, obstacle crossing, fall-prevention exercise, Parkinson's disease and vestibular rehabilitation	IMU, pressure platform, force platform, smartphone inertial sensor, Kinect/depth camera, vibrotactile feedback sensor	COP/COM calculation, time- and frequency-domain sway analysis, individualized sway thresholds, PID or proportional feedback control, BCI composite scoring, virtual-reality/exergame scoring	15	(Pan et al., 2016 , Ma et al., 2015 , Mitternacht et al., 2022 , Allum et al., 2021 , Martins et al., 2020 , Pang et al., 2025 , Lim et al., 2024 , Schwenk et al., 2016a , Jabri et al., 2022 , Schwenk et al., 2016b , van der Logt et al., 2016 , Candreia et al., 2023 , Costantini et al., 2018 , Carpinella et al., 2016 , Martins and Guia, 2020)
**Gait quality, spatiotemporal**	Gait speed, step length, cadence, stride time,	Overground walking, treadmill walking,	IMU, foot sensor, plantar pressure	Heel-strike detection, gait segmentation, ZUPT-based	15	(Ma et al., 2023 , Ma et al., 2018 , Byl et al., 2015 , Kantan et al., 2023 , Sharan et al., 2016 , Pang et al., 2025 , Kim et

**parameters, and symmetry**	stance/swing time, step width, foot progression angle, foot clearance, gait asymmetry, gait rhythm, gait stability, stroke-period symmetry in aquatic exercise	partial weight-bearing gait, gait retraining, post-stroke gait, Parkinson's gait training, aquatic/swimming rehabilitation	sensor, force sensor, motion capture, smartphone sensor, auditory feedback system	foot trajectory estimation, stride-level feature extraction, threshold-based feedback, priority feedback scheme, sonification algorithms, symmetry-ratio calculation		al., 2025 , Schwenk et al., 2016a , Arens et al., 2021 , Schwenk et al., 2016b , van der Logt et al., 2016 , Candreia et al., 2023 , Chen et al., 2017 , Kos and Umek, 2019 , Carpinella et al., 2016)
**Loading, weight distribution, force output, and muscle activation**	Partial weight-bearing compliance, plantar loading distribution, ground reaction force proxy, tibial load, force output, grasp detection, quadriceps activation, EMG amplitude, MVIC-normalized activation	Partial weight-bearing gait, knee-extension training, grasp monitoring, fall-prevention exercise, knee osteoarthritis gait retraining, muscle activation biofeedback	sEMG, accelerometer, IMU, plantar pressure, force-sensitive resistor, force platform, force myography, smart insole	EMG filtering and RMS/MAV extraction, MVC/MVIC normalization, threshold comparison, physics-based force modelling, physics-guided neural network, force/load ratio rules, SVM/LDA classification	13	(Gamecho et al., 2015 , Ma et al., 2023 , Ma et al., 2018 , Byl et al., 2015 , Sengchuai et al., 2022 , Lim et al., 2024 , Sadarangani et al., 2017 , Qi et al., 2025 , Kim et al., 2025 , Kent et al., 2015 , Liu et al., 2025 , Chen et al., 2017 , Martins and Guia, 2020)
**Compensatory movement, postural alignment, and biomechanical error patterns**	Compensatory movement, postural misalignment, abnormal phase progression, trunk/pelvis/knee/foot deviation, lumbar end-range posture, hip-hinge strategy, exercise-specific biomechanical errors	Upper-limb rehabilitation, spine posture control, squat and lunge assessment, low-back-pain rehabilitation, knee rehabilitation exercise, home exercise monitoring	RGB/depth camera, pose-estimation keypoints, IMU, wearable posture sensor, sEMG, mobile app	Error classification, phase progression modelling, PCA reconstruction error, biomechanical rule-based feedback, Grad-CAM or feature-importance visualization, random forest and skeleton-based deep learning	13	(Mennella et al., 2023 , Caviedes et al., 2022 , Zheng et al., 2023 , Jatesiktat et al., 2022 , Heo et al., 2026 , Argent et al., 2018 , AbouGrad et al., 2025 , Arens et al., 2021 , Owlia et al., 2020 , Marusic et al., 2025 , Whelan et al., 2017 , Whelan et al., 2016 , Kent et al., 2015)
**Repetition counting, task completion, and adherence-related performance**	Repetition count, successful repetition, attempted versus successful repetition, hold time, completion rate, exercise frequency, exercise duration,	Home rehabilitation, knee exercise, shoulder exercise, upper-limb training, chair-based exercise, orthopaedic	IMU, mobile app, smartphone sensor, pose-estimation keypoints, wearable motion sensor, cloud platform logs	State-machine repetition counting, peak/threshold detection, app-based success counting, exercise-session logging, automatic repetition categorization, mobile/cloud data synchronization	13	(Gamecho et al., 2015 , Argent et al., 2018 , Chae et al., 2020 , Jarungvittayakon et al., 2025 , Chen et al., 2022 , Sadarangani et al., 2017 , Rawashdeh et al., 2022 , Bevilacqua et al., 2019 , Zhang et al., 2023 , Bell et al., 2019 , Chen et al., 2020 , Argent et al., 2019 , Martins and Guia, 2020)

	training adherence, session logs	rehabilitation, fall-prevention exercise				
**Trajectory similarity, movement pattern similarity, and global quality scoring**	Movement similarity, ROM similarity, trajectory similarity, joint-group distance, target-reaching rate, standard-reaching rate, movement-quality score, similarity to reference performance	Video-based home exercise, posture-estimation rehabilitation tasks, AI-based exercise assessment, markerless motion assessment	RGB/depth video, pose-estimation keypoints, IMU, reference motion capture or standard-template data	Dynamic Time Warping, FastDTW, cosine similarity, MAPE-based similarity, normalized joint-distance calculation, reference-template comparison, pose-fidelity evaluation	8	(Burns et al., 2018 , Zheng et al., 2023 , Jatesiktat et al., 2022 , Heo et al., 2026 , Rawashdeh et al., 2022 , Marusic et al., 2025 , Cunha et al., 2025 , Zhang et al., 2023)

Notes. Categories are not mutually exclusive because a single study could assess multiple movement-quality constructs, such as ROM, exercise correctness, repetition completion, and compensatory movement within the same rehabilitation task. The "No. of studies" column indicates the number of studies mapped to each construct based on the extracted movement-quality indicators and computational methods. Representative study identifiers correspond to the included-study numbering used in the Results section and Supplementary Table 1. ROM, range of motion; IMU, inertial measurement unit; EMG, electromyography; sEMG, surface electromyography; COP, center of pressure; COM, center of mass; MVC, maximum voluntary contraction; MVIC, maximum voluntary isometric contraction; DTW, dynamic time warping; RMSE, root mean square error; ICC, intraclass correlation coefficient; SVM, support vector machine; CNN, convolutional neural network; ST-GCN, spatial-temporal graph convolutional network; ZUPT, zero-velocity update; RMS, root mean square; MAV, mean absolute value; MAPE, mean absolute percentage error; BCI, balance control index.
