# Supplementary material for: Sensor-Based Movement Quality Assessment and Biofeedback for Rehabilitation Exercise: A Scoping Review with Implications for Home-Based and Remote Rehabilitation
Source: Healthcare (Basel). 2026 Aug 7;14(16):2450. doi: 10.3390/healthcare14162450 (PMC13512171; doi:10.3390/healthcare14162450)
Supplement: Supplementary file 1 [file healthcare-14-02450-s001.zip › Supplementary Table S5.pdf]

Supplementary Table S5. Feedback strategies, implementation features, and reported outcomes in the included studies.

Feedback strategy	Feedback recipient	Real-time or remote implementation features	Targeted rehabilitation function	Reported outcomes or evaluation focus	No. of studies	Representative studies
**Visual, screen-based, or app-based feedback**	Patient/user; sometimes therapist	Real-time display through screen, tablet, smartphone app, computer monitor, color-coded target zones, progress graphs, skeleton overlay, movement trajectory, or visual warning	Exercise correction, ROM target achievement, repetition guidance, posture correction, balance control, movement-quality awareness, motivation and engagement	Improved target matching, increased usability and satisfaction, better adherence or exercise completion, improved ROM or functional performance in some clinical studies	34	(Gamecho et al., 2015 , Burns et al., 2018 , Caviedes et al., 2022 , Zheng et al., 2023 , Jatesiktat et al., 2022 , Byl et al., 2015 , Heo et al., 2026 , Argent et al., 2018 , Chae et al., 2020 , Sengchuai et al., 2022 , Jarungvittayakon et al., 2025 , Sharan et al., 2016 , Chen et al., 2022 , Lim et al., 2024 , Qi et al., 2025 , Rawashdeh et al., 2022 , AbouGrad et al., 2025 , Kim et al., 2025 , Schwenk et al., 2016a , Raza et al., 2023 , Jabri et al., 2022 , Bevilacqua et al., 2019 , Schwenk et al., 2016b , Marusic et al., 2025 , Zhang et al., 2023 , Whelan et al., 2017 , Whelan et al., 2016 , Kent et al., 2015 , Liu et al., 2025 , Bell et al., 2019 , Chen et al., 2017 , Chen et al., 2020 , Carpinella et al., 2016 , Argent et al., 2019)
**Auditory feedback or sonification**	Patient/user	Real-time or task-related sound cues, alarm tones, auditory prompts, ecological sound, gait sonification, rhythm-related auditory feedback	Gait retraining, balance training, trunk control, movement timing, exercise correction without requiring continuous visual attention	Feasibility and acceptability of auditory cues; improved or supported gait, balance, or task performance in selected studies; often evaluated through task accuracy, gait parameters, or usability	12	(Ma et al., 2023 , Heo et al., 2026 , Kantan et al., 2023 , Pang et al., 2025 , Kim et al., 2025 , Schwenk et al., 2016a , Owlia et al., 2020 , Schwenk et al., 2016b , Kent et al., 2015 , Costantini et al., 2018 , Liu et al., 2025 , Carpinella et al., 2016)
**Vibrotactile or haptic feedback**	Patient/user	Wearable vibration units, haptic belts, ankle haptic bracelet, vibrotactile cues delivered when sway, gait, or posture parameters exceed predefined thresholds	Balance correction, trunk sway reduction, gait retraining, step-width or foot progression angle adjustment, sensory augmentation	Improved task-specific postural or gait performance in some studies; feasibility of haptic guidance; patient tolerance and device usability reported in selected studies	9	(Pan et al., 2016 , Ma et al., 2015 , Ma et al., 2018 , Allum et al., 2021 , Jabri et al., 2022 , Kent et al., 2015 , van der Logt et al., 2016 , Candreia et al., 2023 , Chen et al., 2017)
**Avatar, virtual reality, game-based, or**	Patient/user; sometimes therapist	Virtual avatar, exergame interface, gamepad-like system, augmented or	Increase engagement, support task-oriented training, improve motor	Improved motivation and usability; some studies reported balance or functional	8	(Gamecho et al., 2015 , Byl et al., 2015 , Heo et al., 2026 , Jarungvittayakon et al., 2025 , Jabri et al., 2022 , Schwenk et al., 2016b , Bell et al., 2019 , Argent et al.,

**interactive feedback**		interactive visual environment, avatar mirroring patient motion	learning, provide intuitive feedback on posture or movement quality	improvements; often assessed through satisfaction, task performance, or clinical measures		2019)
**Therapist-facing feedback and clinician dashboards**	Physiotherapist, physician, clinician, or researcher	Web portal, clinician dashboard, doctor app, therapist interface, cloud-based synchronization, summarized performance metrics, remote review of exercise completion and progress	Remote supervision, treatment monitoring, prescription adjustment, training progression, identification of poor adherence or incorrect exercise execution	Feasibility of remote monitoring, improved clinical visibility of home exercise, user-centred feedback from clinicians, support for individualized rehabilitation management	36	(Byl et al., 2015 , Kent et al., 2015 , Carpinella et al., 2016 , Mennella et al., 2023 , Qi et al., 2025 , Rawashdeh et al., 2022 , Naseer et al., 2025 , AbouGrad et al., 2025 , Raza et al., 2023 , Marusic et al., 2025 , Cunha et al., 2025 , Zhang et al., 2023 , Kantan et al., 2023 , Chae et al., 2020 , Sharan et al., 2016 , Sadarangani et al., 2017 , Jabri et al., 2022 , Arens et al., 2021 , Caviedes et al., 2022 , Ma et al., 2023 , Argent et al., 2018 , Sengchuai et al., 2022 , Jarungvittayakon et al., 2025 , Chen et al., 2022 , Bell et al., 2019 , Chen et al., 2020 , Martins et al., 2020 , Martins and Guia, 2020 , Burns et al., 2018 , Jatesiktat et al., 2022 , Lim et al., 2024 , Whelan et al., 2017 , Whelan et al., 2016 , Kos and Umek, 2019 , Kos et al., 2025 , Mitternacht et al., 2022)
**Mobile health, home-based, or telerehabilitation feedback**	Patient/user and therapist	Smartphone or tablet app, home-based system, remote platform, cloud database, asynchronous feedback, patient progress visualization, therapist review	Home exercise support, adherence monitoring, remote rehabilitation, self-management, continuity of care outside clinical settings	High usability and acceptance in several studies; improved exercise completion or adherence in selected studies; potential for supporting home-based rehabilitation and remote decision-making	14	(Gamecho et al., 2015 , Mennella et al., 2023 , Burns et al., 2018 , Jatesiktat et al., 2022 , Chae et al., 2020 , Jarungvittayakon et al., 2025 , Chen et al., 2022 , Rawashdeh et al., 2022 , AbouGrad et al., 2025 , Jabri et al., 2022 , Cunha et al., 2025 , Bell et al., 2019 , Chen et al., 2020 , Argent et al., 2019)
**Threshold-based or rule-based corrective feedback**	Patient/user; sometimes therapist	Feedback triggered when joint angle, sway, load, EMG, posture, or gait parameters deviate from target thresholds or individualized baseline values	Immediate correction of incorrect movement, ROM targeting, load control, posture stabilization, muscle activation feedback, repetition classification	Commonly reported as feasible and interpretable; often used in real-time systems; clinical effectiveness varied by population and study design	23	(Gamecho et al., 2015 , Pan et al., 2016 , Ma et al., 2015 , Ma et al., 2023 , Sengchuai et al., 2022 , Jarungvittayakon et al., 2025 , Chen et al., 2022 , Pang et al., 2025 , Rawashdeh et al., 2022 , Schwenk et al., 2016a , Jabri et al., 2022 , Arens et al., 2021 , Owlia et al., 2020 , Kent et al., 2015 , van der Logt et al., 2016 , Candreia et al., 2023 , Costantini et al., 2018 , Bell et al.,

						2019 , Chen et al., 2017 , Chen et al., 2020 , Kos and Umek, 2019 , Carpinella et al., 2016 , Martins and Guia, 2020)
**Performance summary, progress monitoring, or adherence feedback**	Patient/user and therapist	Session summaries, exercise logs, completion rate, repetition counts, progress charts, ROM history, cloud-based longitudinal records	Reinforce adherence, enable self-monitoring, support therapist review, inform progression or treatment modification	Improved perceived motivation and self-management; some studies reported higher completion rates or positive user experience; often evaluated through SUS, uMARS, questionnaires, or interview data	13	(Gamecho et al., 2015 , Argent et al., 2018 , Chae et al., 2020 , Jarungvittayakon et al., 2025 , Chen et al., 2022 , Sadarangani et al., 2017 , Rawashdeh et al., 2022 , Bevilacqua et al., 2019 , Zhang et al., 2023 , Bell et al., 2019 , Chen et al., 2020 , Argent et al., 2019 , Martins and Guia, 2020)
**Assessment-only, delayed, or no direct patient feedback**	Researcher, clinician, algorithm developer, or therapist	Offline data processing, post-hoc analysis, delayed review, algorithm validation, movement-quality classification without real-time patient-facing cues	Technical validation, algorithm development, sensor accuracy testing, movement-quality modelling, evidence generation for future feedback systems	Accuracy, validity, reliability, classification performance, model performance, feasibility of future implementation; limited direct evidence for patient behaviour change	12	(Mennella et al., 2023 , Mitternacht et al., 2022 , Jatesiktat et al., 2022 , Sadarangani et al., 2017 , Qi et al., 2025 , Naseer et al., 2025 , AbouGrad et al., 2025 , Kos et al., 2025 , Arens et al., 2021 , Bevilacqua et al., 2019 , Whelan et al., 2017 , Whelan et al., 2016)

Notes. Categories are not mutually exclusive because one system could provide multiple feedback types, such as visual feedback to the patient and progress summaries to the therapist. The “No. of studies” column therefore represents the number of studies mapped to each feedback category rather than mutually exclusive totals. “Assessment-only, delayed, or no direct patient feedback” refers to studies in which sensor data were used primarily for validation, monitoring, or algorithm development without real-time patient-facing corrective feedback. Representative study identifiers correspond to the included-study numbering used in the Results section and Supplementary Table 1. ROM, range of motion; EMG, electromyography; SUS, System Usability Scale; uMARS, user version of the Mobile Application Rating Scale.
