# Supplementary material for: Sensor-Based Movement Quality Assessment and Biofeedback for Rehabilitation Exercise: A Scoping Review with Implications for Home-Based and Remote Rehabilitation
Source: Healthcare (Basel). 2026 Aug 7;14(16):2450. doi: 10.3390/healthcare14162450 (PMC13512171; doi:10.3390/healthcare14162450)
Supplement: Supplementary file 1 [file healthcare-14-02450-s001.zip › Supplementary Table S6 PRISMA_ScR_Checklist.pdf]

Supplementary Table S6. PRISMA-ScR checklist

Preferred Reporting Items for Systematic reviews and Meta-Analyses extension for Scoping Reviews (PRISMA-ScR) Checklist

SECTION	ITEM	PRISMA-ScR CHECKLIST ITEM	REPORTED LOCATION
**TITLE**			
Title	1	Identify the report as a scoping review.	p. 1; Title
**ABSTRACT**			
Structured summary	2	Provide a structured summary that includes (as applicable): background, objectives, eligibility criteria, sources of evidence, charting methods, results, and conclusions that relate to the review questions and objectives.	p. 1; Abstract
**INTRODUCTION**			
Rationale	3	Describe the rationale for the review in the context of what is already known. Explain why the review questions/objectives lend themselves to a scoping review approach.	pp. 2–4; Introduction
Objectives	4	Provide an explicit statement of the questions and objectives being addressed with reference to their key elements (e.g., population or participants, concepts, and context) or other relevant key elements used to conceptualize the review questions and/or objectives.	p. 4; end of Introduction; Section 2.2 Research Questions
**METHODS**			
Protocol and registration	5	Indicate whether a review protocol exists; state if and where it can be accessed (e.g., a Web address); and if available, provide registration information, including the registration number.	p. 4; Section 2.1 Review Design. Protocol retrospectively registered on OSF: https://osf.io/w382t. PRISMA-ScR checklist: Supplementary Table S6
Eligibility criteria	6	Specify characteristics of the sources of evidence used as eligibility criteria (e.g., years considered, language, and publication status), and provide a rationale.	pp. 4–5; Section 2.3 Eligibility Criteria
Information sources*	7	Describe all information sources in the search (e.g., databases with dates of coverage and contact with authors to identify additional sources), as well as the date the most recent search was executed.	p. 5; Section 2.4 Information Sources and Search Strategy; Supplementary Table S2
Search	8	Present the full electronic search strategy for at least 1 database, including any limits used, such that it could be repeated.	p. 5; Section 2.4; full strategies in Supplementary Table S2
Selection of sources of evidence†	9	State the process for selecting sources of evidence (i.e., screening and eligibility) included in the scoping review.	p. 6; Section 2.5 Study Selection
Data charting process‡	10	Describe the methods of charting data from the included sources of evidence (e.g., calibrated forms or forms that have been tested by the team before their use, and whether data charting was done independently or in duplicate) and any processes for obtaining and confirming data from investigators.	p. 6; Section 2.6 Data Charting Process
Data items	11	List and define all variables for which data were sought and any assumptions and simplifications made.	p. 6; Section 2.6 Data Charting Process; Supplementary Table S1
Critical appraisal of individual sources of evidence§	12	If done, provide a rationale for conducting a critical appraisal of included sources of evidence; describe the methods used and how this information was used in any data synthesis (if appropriate).	p. 7; Section 2.8 Critical Appraisal. Formal critical appraisal was not conducted.
Synthesis of results	13	Describe the methods of handling and summarizing the data that were charted.	pp. 6–7; Section 2.7 Evidence Categorization and Data Synthesis
**RESULTS**			
Selection of sources of evidence	14	Give numbers of sources of evidence screened, assessed for eligibility, and included in the review, with reasons for exclusions at each stage, ideally using a flow diagram.	pp. 7–8; Section 3.1 Study Selection; Figure 1
Characteristics of sources of evidence	15	For each source of evidence, present characteristics for which data were charted and provide the citations.	pp. 8–14; Sections 3.2–3.4; Tables 1–2; Supplementary Tables S1 and S3
Critical appraisal within sources of evidence	16	If done, present data on critical appraisal of included sources of evidence (see item 12).	Not applicable. Formal critical appraisal was not conducted; see p. 7, Section 2.8.
Results of individual sources of evidence	17	For each included source of evidence, present the relevant data that were charted that relate to the review questions and objectives.	Supplementary Table S1; summarized in pp. 8–16, Sections 3.2–3.6
Synthesis of results	18	Summarize and/or present the charting results as they relate to the review questions and objectives.	pp. 8–16; Sections 3.2–3.6; Figures 2–4; Tables 1–2; Supplementary Tables S3–S5
**DISCUSSION**			
Summary of evidence	19	Summarize the main results (including an overview of concepts, themes, and types of evidence available), link to the review questions and objectives, and consider the relevance to key groups.	pp. 17–26; Sections 4.1–4.6
Limitations	20	Discuss the limitations of the scoping review process.	pp. 26–27; Section 4.7 Strengths and Limitations of This Review
Conclusions	21	Provide a general interpretation of the results with respect to the review questions and objectives, as well as potential implications and/or next steps.	p. 27; Section 5 Conclusions
**FUNDING**			
Funding	22	Describe sources of funding for the included sources of evidence, as well as sources of funding for the scoping review.	p. 28; Funding statement

SECTION	ITEM	PRISMA-ScR CHECKLIST ITEM	REPORTED LOCATION
		Describe the role of the funders of the scoping review.	

Note. Page numbers refer to the rendered version of Manuscript_V7 used to complete this checklist. Section numbers and Supplementary Table numbers are provided to support verification if pagination changes during editorial processing.

JBI = Joanna Briggs Institute; PRISMA-ScR = Preferred Reporting Items for Systematic reviews and Meta-Analyses extension for Scoping Reviews; OSF = Open Science Framework.

Source: Tricco AC, Lillie E, Zarin W, O'Brien KK, Colquhoun H, Levac D, et al. PRISMA Extension for Scoping Reviews (PRISMA-ScR): Checklist and Explanation. Ann Intern Med. 2018;169:467–473. doi: 10.7326/M18-0850.
